# Harnessing copper–strontium synergy in an injectable composite for multi-pathway bone regeneration

**DOI:** 10.1039/d6ra01689h

**Published:** 2026-05-13

**Authors:** Zhengqing Zhu, Azin Khodaei, Helen E. King, Harrie Weinans, Saber Amin Yavari

**Affiliations:** a Department of Orthopedics, University Medical Center Utrecht Utrecht 3508GA The Netherlands S.AminYavari@umcutrecht.nl; b MARUM, Faculty of Geosciences, University of Bremen Bremen 28359 Germany; c Department of Earth Sciences, Faculty of Geosciences, Utrecht University Utrecht 3584CB The Netherlands; d Regenerative Medicine Centre Utrecht, Utrecht University Utrecht 3508GA The Netherlands

## Abstract

Bone regeneration, particularly in critical-sized defects, remains a significant clinical challenge. Hydroxyapatite (HA) is widely used due to its similarity to bone mineral, yet its biological activity often requires improvement. Incorporation of therapeutic metal ions has shown promise, but the combined and mechanistically distinct effects of copper (Cu) and strontium (Sr) remain underexplored. Cu and Sr influenced stem cells *via* different signaling pathways: Cu enhanced angiogenesis and modulated differentiation, while Sr promoted osteogenesis and bone remodeling. Importantly, these differences extended beyond stem cell regulation; Cu and Sr activated distinct mechanisms in both osteogenesis and angiogenesis. When combined, this dual action resulted in a true synergistic effect, independently enhancing osteogenic and angiogenic outcomes beyond what either ion can achieve alone. To exploit this, we developed an injectable dual-network hydrogel composed of polyvinyl alcohol (PVA) and sodium alginate (SA), incorporating Cu/Sr co-doped HA synthesized *via* hydrothermal methods. By varying synthesis temperature (80, 120, 160 °C), we revealed the dependence of HA crystallinity on bioactivity and identified 120 °C as optimal for coupling mechanical stability with biological performance. Cytotoxicity assays established the safe Cu threshold and defined the single-ion optima for Cu and Sr. Strikingly, co-doping at these optima yielded a composite with excellent injectability, robust *in vitro* and *ex vivo* biocompatibility, and, most importantly, synergistic promotion of both osteogenesis and angiogenesis through complementary mechanisms. This dual-ion strategy provides a novel route for synchronizing bone and vascular regeneration in bone repair.

## Abstract 2

Fabrication of an injectable PVA/alginate dual-network hydrogel loaded with Cu/Sr-doped hydroxyapatite (HA). (A) Synthesis of Cu/Sr-HA. (B) Mechanism of crosslinking. (C) The composite promotes angiogenesis and bone regeneration.

## Graphical abstract

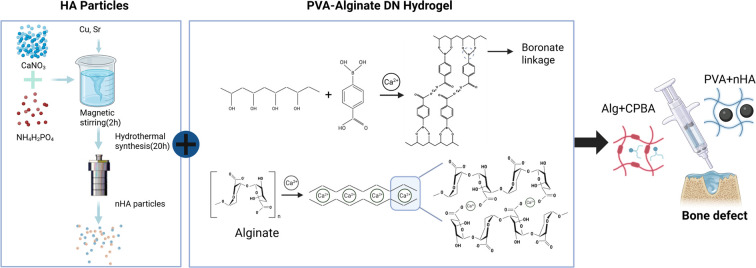

## 1. Introduction

Bone regeneration remains a critical challenge in regenerative medicine and orthopedics,^1–3^ particularly for critical-size defects (1–3 cm)^4,5^ that exceed the body's innate healing capacity.^6,7^ These defects caused by trauma, tumor resections, or degenerative disorders, often require grafts to bridge gaps and stimulate repair.^8^ Autografts (patient-derived bone) are considered the gold standard for their osteogenic activity and compatibility, yet their use is constrained by donor site morbidity and a limited supply. Allografts (cadaveric bone) avoid donor site concerns, though immune rejection and disease spread are still possible, and variable mechanical properties. Xenografts (animal-derived bone) face similar immunological barriers and heightened infectious disease risks.^9^ While these graft-based approaches dominate clinical practice, their limitations; including secondary surgeries, biological variability, and infection risks; drive demand for synthetic alternatives with tunable properties and reduced complications.^9–12^

Synthetic bone grafts, such as hydroxyapatite (HA), address autografts limitations by offering tunable mechanical properties and eliminating donor site morbidity.^13–15^ While HA's chemical similarity to natural bone minerals underpins its osteoconductivity, its biological performance hinges on its crystallinity-a parameter controllable through hydrothermal synthesis conditions.^16^ High-crystallinity HA, synthesized at elevated temperatures (>200 °C), exhibits robust mechanical strength suitable for load-bearing applications but demonstrates slow degradation rates that hinder ionic exchange and new bone formation.^17^ Conversely, HA produced at lower temperatures (80–120 °C) has a lower crystallinity that mimics nascent bone minerals more effectively, enhancing bioresorption and osteoblast adhesion. However, excessive amorphous content produced at lower synthesis temperatures compromises structural integrity, risking premature graft dissolution.^18^ Hydrothermal synthesis enables precise crystallinity modulation by adjusting reaction temperatures.^19^ Feng *et al.* demonstrated that increasing temperatures from 80 °C to 200 °C shift HA crystal growth from *c*-axis to *a*-axis dominance, altering surface topography and protein adsorption dynamics.^20^ Balasundaram *et al.* showed that amorphous calcium phosphate supports greater osteoblast adhesion than nanocrystalline or sintered HA, though their extreme temperature differentials (80 °C *vs.* 1100 °C) complicate direct crystallinity comparisons.^17^ Emerging multi-temperature hydrothermal protocols proposed gradient crystallinity scaffolds, which potentially enabled spatially controlled degradation and ossification.

Given the current state of bone tissue engineering, there is a growing shift away from expensive and potentially risky recombinant growth factors (such as BMP-2 and VEGF) toward more stable, ‘drug-free’ ion-based approaches.^21^ In this context, non-metallic elements like calcium and phosphorus are essential for forming the structural mineral matrix, whereas metallic trace elements (*e.g.*, Cu, Sr, Mg, Zn) have emerged as powerful bio-instructive cues that can actively regulate angiogenesis, osteogenesis, and bone remodelling.^22^ HA exhibits unique ion-exchange capabilities, enabling the substitution of surface calcium ions (Ca) with therapeutic divalent metals such as copper (Cu) and strontium (Sr) while preserving its crystalline structure.^23^ This property allows the functionalization of HA biomaterials to enhance specific biological responses during bone regeneration. Cu demonstrated multifunctional roles, serving as a cofactor for enzymatic proteins involved in electron transfer, oxygen transport, and metal homeostasis.^24^ Its incorporation into bone grafts promoted angiogenesis through vascular endothelial growth factor activation, improving nutrient delivery to implantation sites.^25^ Additionally, Cu exhibited antimicrobial effects and stimulated collagen deposition, although optimal concentrations remained critical.^26^ Studies indicated dose-dependent effects, where low Cu levels (≤1.0 wt%) enhanced osteoblast activity and vascularization, while higher concentrations (≥2.0 wt%) impaired alkaline phosphatase activity, collagen synthesis, and ectopic bone formation in rodent models.^27^ This biphasic response underscored the necessity for precise dopant control during material synthesis.^28^ In contrast, Sr exhibits dual regulation of bone remodeling,^29^ including stimulation of osteoblast differentiation through calcium-sensing receptor (CaSR) activation and suppression of osteoclast-mediated resorption.^30^ Its ionic radius and similarity to calcium facilitate integration into the atomic structure of HA and subsequent release during material degradation.^31^ Clinically, strontium ranelate has demonstrated efficacy in osteoporosis treatment since 2004, validating its anabolic and anti-catabolic mechanisms.^31^ Sr-doped HA coatings on orthopedic implants showed increased osseointegration through upregulation of osteogenic markers such as RUNX2 and osteocalcin^30^. Emerging research explored the synergistic effects of multi-ion doping, though available evidence remains limited. While Wu *et al.*^25^ Demonstrated enhanced osteogenesis with Cu–Si co-doping, there remains a critical need to systematically investigate the specific combination of Cu and Sr. Unlike Si, which mainly supports early matrix synthesis, Sr is distinguished by its unique dual-action pharmacological profile.

Previous studies highlighted the osteogenic and angiogenic benefits of Cu- or Sr-doped HA.^32,33^ Cu enhances vascularization and osteogenic differentiation, while Sr promotes bone formation and mineral density. However, research into the combined effects of Cu and Sr co-doping in HA, especially in hydrogel systems, is limited. Careful control of the metal ion dosage is essential, as excessive amounts can inhibit osteogenesis and cause inflammation, oxidative stress, or systemic toxicity.^34^ There are no established toxic thresholds for Cu and Sr, and the influence of HA crystallinity on biocompatibility and cell attachment in co-doped systems remains underexplored. Addressing these gaps could help optimize bone graft materials for both mechanical and biological performance. To address these challenges, a dual-network hydrogel combining polyvinyl alcohol (PVA) and sodium alginate (SA) with Cu- and Sr-doped HA has been developed here. PVA offers excellent biocompatibility and mechanical strength, forming stable hydrogels through its hydroxyl groups.^35^ 4-Carboxyphenylboronic acid (CPBA) acts as a biocompatible cross-linker, creating covalent boronate ester bonds with PVA.^36^ SA, a natural polysaccharide, further enhances hydrogel stability and cell attachment by forming an ionic network of calcium ions.^37^ Hydrothermally synthesized Cu and Sr co-doped HA further reinforces the hydrogel by enhancing its mechanical strength and providing controlled release of bioactive ions. This study introduces a composite of PVA, SA, Cu/Sr-doped HA, and evaluates how hydrothermal synthesis conditions influence HA crystallinity and, in turn, affect the mechanical and biological properties of the composite. In addition, the study explores the cytotoxicity thresholds and differentiation properties of Cu and Sr within the composite. Finally, it also systematically investigates the impact of Cu and Sr co-doping at different concentrations on HA osteogenic and angiogenic potential.

## 2. Materials and methods

### 2.1 Materials

Calcium nitrate (CaNO_3_, MW: 236.15, CAS: 13477-34-4), copper nitrate (CuNO_3_, MW: 241.60, CAS: 10031-43-3), strontium nitrate (SrNO_3_, MW: 211.63, CAS: 10042-76-9), ammonium hydrogen phosphate (MW: 115.03, CAS:7722-76-1), ammonium hydroxide (maximum 33% NH_3_), sodium alginate (SA, CAS: 9005-38-3), Polyvinyl alcohol (PVA, MW: 85 000-124,000, CAS: 9002-89-5), and calcium chloride (CaCl_2_, MW: 110.98, CAS: 10043-52-4) were purchased from Sigma Aldrich, Germany. 4-Carboxyphenylboronic acid (CPBA, CAS: 14047-29-1) was purchased from Thermo-Fisher Scientific Chemicals, USA. Advanced DMEM (Thermo-Fisher Scientific, USA), endothelial cell growth medium-2 BulletKit (EGM-2, CC-3162, Lonza, Switzerland), RPMI 1640 culture medium (Thermo-Fisher Scientific, USA), penicillin-streptomycin antibiotic (Pen-Strep, Thermo-Fisher Scientific, USA), l-ascorbic acid 2-phosphate sesquimagnesium salt hydrate (l-ascorbic acid, A8960, Sigma Aldrich, Germany), Dexamethasone (DXMS, D8893, Sigma Aldrich, Germany), β-glycerophosphate disodium salt hydrate (BGP, G9422, Sigma Aldrich, Germany) and fetal bovine serum (FBS, Invitrogen, UK) were purchased for *in vitro* cell culturing. Resazurin sodium salt (MW: 251.17, CAS: 62758-13-8, Sigma Aldrich, Germany), phorbol 12-myristate 13-acetate (PMA, P8139, Sigma Aldrich, Germany), Pico Green reagent (P7589, Thermo-Fisher Scientific, USA), and live/dead Kit (Thermo-Fisher Scientific, USA) were purchased for cell viability test. The Matrigel (356234, Corning, USA) was purchased for the tube formation assay. Alkaline Phosphatase (ALP) kit (CAS: 9001-78-9), Alizarin-Red S (MW: 342.26, CAS: 130-22-3), and Cetylpyridinium chloride (CPC, MW: 358.00, CAS: 6004-24-6) were purchased from Sigma Aldrich, Germany, for osteogenesis assessment. Trizol (Thermo-Fisher Scientific, USA), iScript cDNA synthesis kit (Bio-Rad, USA), and iTaq Universal SYBR Green Supermix (Bio-Rad, USA) were purchased for isolation of RNA, synthesis of cDNA, and qRT-PCR assay. Lipoploysaccharide (LPS, 00-4976-03, ThermoFisher Scientific, USA) and Tumor Necrosis Factor-alpha (TNF-α, DY210, RδD system, USA) ELISA kits were purchased for basic whole blood assay.

### 2.2 Methods

#### 2.2.1 Synthesis of HA and metal-doped HA particles

The hydrothermal synthesis method was used to synthesize HA with different crystallinity.^38^ Briefly, an equal volume of 1 M calcium nitrate was slowly dropped (1.5 ml min^−1^) into 0.6 M ammonia hydrogen phosphate solution ([Ca]/[P] was 1.67), and then the pH value was adjusted to 10 using 25% ammonium hydroxide, and the mixture was stirred magnetically for two hours at room temperature. The resulting suspension was then transferred into a stainless-steel autoclave with an inner liner of polytetrafluoroethylene, where the volume of the solution was 70% of the autoclave capacity. Several experiments were run where the autoclave was heated to 80 °C, 120 °C, or 160 °C for a duration of 20 h. The precipitate was collected and vortexed with ethanol homogenously, after which the precipitate was separated from the ethanol *via* centrifugation at 2000 rpm, and the procedure was repeated 3 times for each precipitate. This procedure was then repeated with Milli Q water. Following the washing procedure, the precipitation was uniformly dispersed in Milli Q water using ultrasonication before being lyophilized. After lyophilization, the HA powder was stored at room temperature in a sealed plastic tube prior to analysis and further testing.

The formation of HA is based on the following reactions:10Ca(NO_3_)_2_ + 6(NH_4_)_2_HPO_4_ + 8NH_4_OH → Ca_10_(PO_4_)_6_(OH)_2_ + 6H_2_O + 20NH_4_NO_3_

For the synthesis of metal (M)-doped HA, Ca was replaced by different molar ratios of Cu (0.5%, 1%, 3%, 5%) and Sr (2%, 4%, 8%, 12%). These materials will be coded M-HA. The molar ratio of [Ca + M]/[P] was kept at 1.67. The synthesis process of the dual metal ions Cu/Sr-HA was identical to the described methods above, with the addition of Cu and Sr (0.5 mol% Cu/4 mol% Sr, or 1 mol% Cu/4 mol% Sr) to the mother solution during the synthesis of HA. The hydrothermal synthesis method and powder acquisition were similar to those described above.

#### 2.2.2 Synthesis of HA-PVA/SA injectable composites

Solution A was prepared by dissolving PVA, 14wt% PVA was stirred in Milli Q water at 80 °C for 3 hours until the solution became homogeneous and transparent. For solution B, 6wt% SA was dissolved in Milli Q water by stirring for 2 hours at room temperature. Different concentrations of CPBA solution in 10wt% NaOH was prepared, and the pH of the solution was adjusted (7.4 or 8.4) using 1 M HCl (solution C). To prepare the composites, initially, solutions A, B were mixed in the presence of 30%wt HA or M-HA particles; thereafter, solution C was added to the mixtures to crosslink the PVA network. Finally, the forming gel was rinsed with 1.4 M CaCl_2_ solution for ionic crosslinking of SA networks to form a double network. The final cylindrical samples were created with a 6 mm biopsy punch (*D* = 6 mm, *H* = 3 mm). To study composites in the dry state, the samples were lyophilized and kept at room temperature.

#### 2.2.3 HA-PVA/SA composites characterization

The synthesized HA particles were examined by scanning electron microscopy and energy-dispersive X-ray spectroscopy (SEM, Zeiss EVO 15-EDX, Bruker Xflash 6/30 dual detector) to assess morphology. Phase composition was analyzed by X-ray spectroscopy (XRD, Bruker D8 Advance, Cu Kα, *λ* = 0.15406 nm) using a 0.02° step size.

The crystallite size was calculated using the Scherrer (*D*_s_) equation:
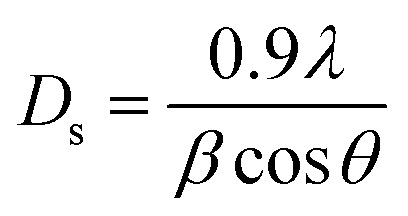


The *β* represents the full width at half maximum (FWHM); the (002) plane was chosen to calculate *D*_s_ for all synthetic HA samples.^39^

A Thermo Scientific Nicolet 6700 Fourier transform Infrared spectrometer (FTIR) equipped with a Pike GladiATR attenuated total reflectance (ATR) unit with diamond window was recorded in the 400 to 4000 cm^−1^ range to investigate molecular interactions. A WITec alpha 300 R confocal Raman microscope equipped with a 532 nm laser and 600 grooves per mm grating was used to obtain the spectra of HA particles to look into the effect of different dopants in the structure of HA. Each spectrum was acquired for 2 seconds 10 times to obtain spectra with good signal to noise ratios. According to the Raman spectroscopy results, the area ratio of the characteristic *v*_1_ phosphate band (957 cm^−1^) to OH/water band (3572 cm^−1^), as well as the FWHM of the 3572 cm^−1^ band, were systematically analyzed for the HA, 1% Cu-HA, and 5% Cu-HA particles to investigate the structural changes associated with ion incorporation into HA.

#### 2.2.4 *In vitro* experiments

##### 2.2.4.1 Acquisition and cultivation of human bone marrow mesenchymal stem cells (hMSCs)

Based on the approval of the institutional medical ethics committee (METC 08-001 K and METC 07-125 C protocols), healthy bone marrow samples were obtained from patients undergoing orthopedic surgery at the University Medical Centre Utrecht, the Netherlands. Human material was obtained in accordance with the Declaration of Helsinki, with the approval of the local medical ethical committee (University Medical Center Utrecht (UMCU), Utrecht, The Netherlands) under the protocols METC 08-001 K and METC 07-125 C, and with the written consent of the participants. A mononuclear cell fraction was obtained by Ficoll–Paque centrifugation. Subsequent cultures were grown in advanced DMEM supplemented with 100 units mL^−1^ of Pen-Strep and 10% FBS until the cells fully adhered to the bottom and proliferated to 70–80% confluence. The adherent primary cells were then passaged to the corresponding generation. The 4th or 5th passage cells were used for osteogenic differentiation experiments, while the 5th or 6th passage cells were used for cytotoxicity and cell adhesion assays. These hMSCs were maintained in a humidified incubator (37 °C, 5% CO_2_, MCO-170AICUV-PE, Panasonic). The medium was refreshed twice a week during the incubation period.

##### 2.2.4.2 Cultivation and expansion of human umbilical vein endothelial cells (HUVECs)

HUVECs (Lonza, Switzerland) were cultured in PBS-gel-coated flasks. To pre-coat t175 flasks (Greiner Bio-One, Germany), 10 ml PBS-gel (1% gelatine) was incubated in the flasks for 5–6 min in incubator conditions. Then, HUVECs were expanded in EGM-2 medium and were used in passages 6 to 8.

##### 2.2.4.3 Cultivation and differentiation of THP-1 cell line

THP-1 human monocytic leukemia cells (R&D Systems, USA) were maintained in RPMI-1640 supplemented with 10% (v/v) FBS and 1% (v/v) Pen-Strep. For THP-1 differentiation into M0 macrophages, 5 × 10^5^ cells per ml were incubated in 100 nM PMA medium for 24 h till macrophages adhered to the bottom.

##### 2.2.4.4 Samples preparation for *in vitro* assays

Released supernatant from the composites was collected for *in vitro* assays. In short, the composites were immersed in advanced DMEM in a six-well plate and incubated at 37 °C with 5% CO_2_, the mass-to-volume ratio is 1g/10 mL (based on ISO 10993-13). The supernatant was then collected and refreshed after 1, 3, and 7 days of incubation.

*In vitro* assays were performed following supernatant replacement on days 0, 2, and 6, with the supernatant collected after 1, 3, and 7 days of medium immersion. To prepare the supernatant for osteogenic differentiation experiments, advanced DMEM was replaced with osteogenic induction medium during composite incubation. After day 7, the regular osteogenic induction medium changes continued. To prepare the supernatant for angiogenesis assays, EGM-2 medium was substituted during composite incubation and cell culture.

##### 2.2.4.5 Cytotoxicity

Alamar blue assay was performed to investigate the metabolic activity of cells of the composites. Briefly, 5.54 mg resazurin salt was dissolved in 50 ml PBS. The mixed solution was filtered using a 0.22 µm filter and then diluted 10 times in advanced DMEM. 5 × 10^3^ hMSCs were seeded in each well of a 96-well plate and cultured with the supernatant of composites for 1 and 7 days, then incubated with the 200 µL diluted resazurin salt solution in the cell incubator for 2 h. After that, 100 µL supernatant of each group was transferred to a new 96-well plate for the microplate reader (excitation: 560 nm, emission: 590 nm, BMG LABTECH, Germany). The data collected from all experimental groups were normalized against the positive control (cells incubated in regular medium), and the threshold of 70% metabolic activity was used to determine cytotoxicity according to ISO 10993-5:2009. Pico Green assay was performed on lysed cells to quantify DNA (dsDNA) content in each well. The effect of HA crystallinity on cell adhesion was also evaluated using composites with HA synthesized at the different temperatures (80 °C, 120 °C, and 160 °C). In short, when the composites (3 mm × 3 mm) were settled in a 96-well suspension plate, the hMSCs were then seeded on top of the composites' surface for 1 day; followed by performing the Alamar blue and Pico Green assays as described above. Cell viability after 1 and 7 days of incubation was visually analyzed by a fluorescent staining live/dead Kit. Live cells were stained with Calcein AM (green), whereas dead cells were stained with ethidium homodimer-1 (red). The imaging was performed using Confocal Laser Scanning Microscopy (CLSM, Leica SP8X, Germany). The supernatant was collected from the composites with (0.5%, 1%, 3%, 5%) Cu and (2%, 4%, 8%, 12%) Sr-HA particles to investigate the biocompatibility of different concentrations of metal ions. Alamar blue, Pico Green assay, and live-dead staining were conducted to evaluate the initial cell viability and proliferation on day 1 and day 7.

##### 2.2.4.6 HUVECs migration and tube formation assays

For the migration assay, HUVECs were seeded in 12-well plates at a density of 5 × 10^5^ cells and incubated for 24 h until the cells became overconfluent. Subsequently, a scratch was made on each well with a sterile P200 pipette tip, and after washing with PBS, the unattached HUVECs were removed. The adherent HUVECs were then co-incubated with the supernatant medium of composites for 0 and 24 h, and HUVECs were imaged by an inverted microscope (OLYMPUS, Japan). Cell migration rate was quantified as the fraction of the closed area and the initial wound area using ImageJ software.

For the tube formation assay, A volume of 30 µL thawed Matrigel was placed in a pre-cooled 96-well plate and left at 37 °C for 1 h to form a gel. Subsequently, HUVECs were seeded on Matrigel at a density of 2 × 10^4^ cells per well. They were incubated with the released supernatant for 6 h at 37 °C. The tube formation was observed with an optical microscope (DMI1, Leica, Germany), and the number of nodes, junctions, branches, branch length, and tube length was quantified by an angiogenesis analyzer plugin automatically in ImageJ software.

##### 2.2.4.7 ALP and alizarin red assays

For the ALP assay, 5 × 10^3^ hMSCs were seeded in 96-well plates for 24 h incubation, then the released supernatants were added to refresh the medium. Cells were collected and characterized after 7, 10, and 14 days. Cells were permeabilized with 0.2% Triton X-100 for 30 minutes, and the ALP kit was performed at room temperature according to the ALP kit instructions.

To investigate mineralization of the extracellular matrix, 5 × 10^4^ hMSCs were seeded in a 48-well plate and incubated with released supernatant and regular osteogenic medium. At day 21, we fixed the cells in 100% ethanol for 15 minutes, followed by a 30-minute stain with 0.2% Alizarin Red S (pH 4.2). After imaging the wells with a stereomicroscope (SZ61, OLYMPUS, Japan), the attached alizarin red dye was dissolved in 10% CPC in 10 mM Na_2_H_2_PO_4_ at pH 7.2. The concentration of the Ca ions was quantified based on the concentration of the adhered dye.

##### 2.2.4.8 RNA extraction and qRT-PCR analysis

mRNA expression of osteogenic (RUNX2/ALP/Col1/OCN) and angiogenic (CD31/VEGF) signatures was determined *via* qRT-PCR. GAPDH and YWHAZ were used as housekeeping genes. The sequence of primers used is shown in Table 1. After 14 days, the cells were collected with a cell scraper (541070, Greiner Bio-One, Germany) and the total RNA of the cells was extracted by using the Trizol method. The extracted 1 µg RNA was reverse transcribed to 20 µL cDNA by an iScript cDNA synthesis kit, then the cDNA was diluted to 250 µL. The next amplification-reaction system's total volume was 15 µL, comprising 5 µL cDNA, 2.5 µL diluted primers, and 7.5 µL iTaq Universal SYBR Green Supermix. The denaturation was at 95 °C for 15 s, and annealing/extension at 60 °C for 30 s, the conditions were 40 cycles, while for the YWHAZ gene, 65 °C was set as the annealing/extension temperature. Target mRNA expression was quantified by the 2^−ΔΔCt^ method; the control group was normalized and then contrasted with the other groups.

**Table 1:** Primer sequences utilized in the qRT-PCR assay

Target	Primer sequence (5′–3′)
Human-GAPDH-FW	CAAGATCATCAGCAATGCCT
Human-GAPDH-RV	CAGGGATGATGTTCTGGACAG
Human-YWHAZ-FW	ACTTTTGGTACATTGTGGCTTCAA
Human-YWHAZ-RV	CCGCCAGGACAAACCAGTAT
Human-RUNX2-FW	ATGCTTCATTCGCCTCAC
Human-RUNX2-RV	ACTGCTTGCAGCCTTAAAT
Human-ALP-FW	TCTTGGGGTGCACCATGATT
Human-ALP-RV	ATTCAGTGTCTCTTGCGCTTG
Human-Col1a1-FW	TCCAACGAGATCGAGATCC
Human-Col1a1-RV	AAGCCGAATTCCTGGTCT
Human-OCN-FW	CCTCACACTCCTCGCCCTAT
Human-OCN-RV	GCTTGGACACAAAGGCTGCAC
Human-CD31-FW	AGTCCAGATAGTCGTATGTGAAATG
Human-CD31-RV	TACCGCAGGATCATTTGAGTTC
Human-VEGFA-FW	CACCATGCAGATTATGCGGATCAAAC
Human-VEGFA-RV	GCTCCAGGACTTATACCGGGATTTC

#### 2.2.5 Basic whole blood assay

Two healthy donors' blood was obtained *via* the UMC Utrecht Mini Donor Dienst, with written informed consent and authorization from the local medical ethics board. Ethical approval was granted by the Medical Ethics Committee of the University Medical Center Utrecht (METC protocol 07-125 C; 1 March 2010). 10 ml of blood was diluted 5× in RPMI medium and then incubated with the composites in a 48-well plate at 37 °C and 5% CO_2_ for 24 h. Diluted blood with 1 ng ml^−1^ LPS and 25 ng ml^−1^ PMA groups were used as positive controls. Eventually, the blood from each well was centrifuged (500 g, 5 min) to collect serum for TNF-α quantification using an ELISA kit.

#### 2.2.6 Statistical analysis

Three technical replicates were considered for all quantitative measurements. All the data were graphed and analyzed using GraphPad Prism 8, ImageJ, and Origin 2021. The values were reported as mean ± standard deviation (SD) in all the graphs. Statistical analysis employed one-way and two-way ANOVA (for grouped comparisons), and multiple comparisons tests were corrected using Bonferroni statistical hypothesis testing. All results were analyzed in terms of statistically significant differences for *p < 0.05, **p < 0.01, ***p < 0.001.

## 3. Results

### 3.1 Characterization

**Fig. 1:**
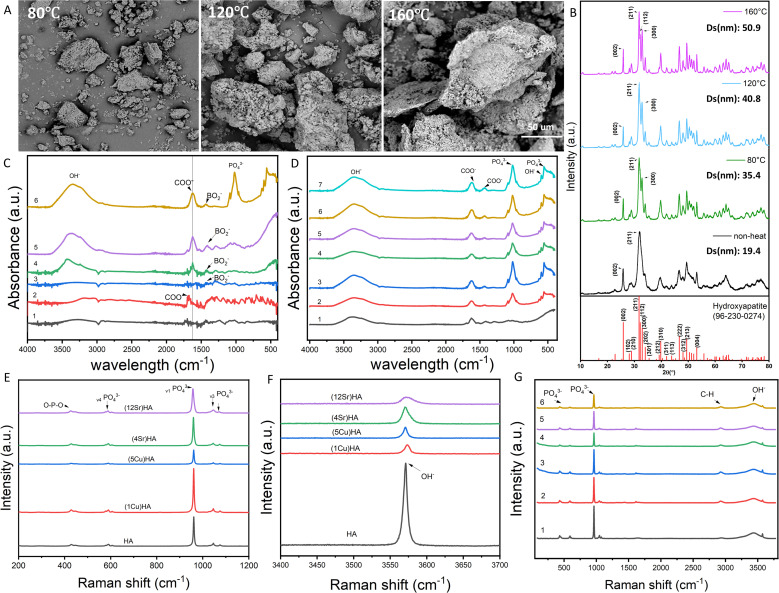
Characterizations of synthesized HA particles and co-doped Cu/Sr-HA composites. (A) SEM images illustrating the morphological variations of HA particles synthesized at 80 °C, 120 °C, and 160 °C. Scale bar: 50 µm. (B) XRD patterns obtained from HA synthesized at different temperatures (room temperature, 80 °C, 120 °C, and 160 °C), and the comparison of HA with the database of standard HA 96-230-0274 (*D*_s_ calculated by Scherrer's equation using the (211) peak). (C) ATR FTIR spectra of hydrogel components (PVA, SA) and their combinations. The small negative band at 2950 cm^−1^ resulted from an isopropanol contaminant in the background spectrum visible from materials with a low absorbance; it does not affect the rest of the spectrum (1: PVA, 2: CPBA, 3: PVA + CPBA, 4: PVA + CPBA + Ca, 5: PVA + CPBA + SA + Ca, 6: PVA + CPBA + SA + Ca + HA). (D) FTIR spectra of co-doped Cu/Sr-HA composites (1: PVA + CPBA + SA + Ca, 2: PVA + CPBA + SA + Ca + HA, 3:PVA + CPBA + SA + Ca +4% Sr-HA, 4: PVA + CPBA + SA + Ca +0.5Cu%-HA, 5: PVA + CPBA + SA + Ca +1Cu%-HA, 6: PVA + CPBA + SA + Ca +0.5% Cu/4% Sr-HA, 7: PVA + CPBA + SA + Ca +1% Cu/4%Sr-HA). (E and F) Raman spectra of HA particles doped with different concentrations of Cu (0.5%, 1%, 5%) and Sr (4%, 12%). (E) Raman shift: 200–1200 cm^−1^. (F) Raman shift: 3400–3700 cm^−1^. (G) Raman spectra of the co-doped Cu/Sr-HA composites (1: PVA + CPBA + SA + Ca + HA, 2: PVA + CPBA + SA + Ca +4% Sr-HA,3: PVA + CPBA + SA + Ca +0.5 Cu%-HA, 4: PVA + CPBA + SA + Ca +1Cu%-HA, 5: PVA + CPBA + SA + Ca +0.5% Cu/4% Sr-HA, 6: PVA + CPBA + SA + Ca +1% Cu/4% Sr-HA).

At the 80 °C synthesis condition, the HA particles were relatively small and exhibited irregular morphology, with visible agglomerations. At 160 °C, the SEM images revealed a significant increase in particle size with a more compact and agglomerated morphology, suggesting that higher synthesis temperatures lead to increased aggregation of HA particles (Fig. 1A). XRD analysis of HA synthesized at various temperatures (non-heated, 80 °C, 120 °C, and 160 °C) shows distinct changes in crystallinity and peak definition (Fig. 1B). The standard HA shows sharp and well-defined peaks, characteristic of a highly crystalline material. Peaks at specific 2*θ* values such as 25°, 31.8°, 32°, and 32.9° correspond to the (002), (211), (112), and (300) planes of crystalline HA (database: 96-230-0274,^40,41^ respectively. In the non-heated group, synthesized at room temperature, all diffraction peaks exhibited significant broadening. Recent crystallographic work by Kis *et al.*^42^ Suggests that native bone minerals exist as monoclinic calcium phosphate mesocrystals. It is highly plausible that our poorly crystalline, non-heated precipitate initially forms as a similar monoclinic-like precursor phase. The subsequent application of hydrothermal heat treatment (80–160 °C) provided the necessary thermodynamic driving force for continuous crystal growth and structural maturation. There is a noticeable improvement in crystallinity with sharper and more defined peaks in 80 and 120 °C patterns. The sample synthesized at 160 °C showed the sharpest and most defined peak among the synthesized samples, closely resembling the standard HA. The lines became sharper and the *D*_s_ size became bigger as the temperature increased. Higher synthesis temperatures promote higher crystal growth and order within the HA structure, as evidenced by the sharpening of XRD peaks.^38^

To systematically elucidate the effect of specific ion doping on the crystallographic structure, XRD phase analysis was performed on all single-doped (1Cu, 5Cu, 4Sr, 12Sr) and the optimal co-doped (1Cu/4Sr) HA powders synthesized at 120 °C (SI Fig. S4). All doped samples exclusively exhibited the characteristic diffraction peaks of the standard HA phase, with a complete absence of secondary metal oxide phases (*e.g.*, CuO or SrO). Furthermore, while the incorporation of Cu and Sr ions inevitably led to a slight alteration in the HA crystallinity, the extent of this change remained highly limited. Quantitative evaluation of the (002) reflection using the Scherrer equation revealed a systematic dopant-induced crystallite refinement. The crystallite size (*D*_s_) decreased from 40.8 nm (pure HA) to 38.8 nm for the optimal 1Cu/4Sr-HA, and further down to ∼37.0 nm for the heavily doped 5Cu and 12Sr groups.^43,44^ And the characteristic diffraction peaks of the 12Sr-HA exhibited a discernible shift toward lower 2*θ* angles compared to pure HA. This leftward shift directly indicates an increase in the interplanar *d*-spacing and an expansion of the unit cell dimensions.^44^

The morphology and elemental distribution of (1%, 5%) Cu or (4%, 12%) Sr-HA were analyzed using SEM-EDX (SI Fig. S1A). The elemental mapping of Ca, phosphorus, Cu, and Sr indicated that these elements were uniformly distributed throughout the HA crystals, demonstrating successful doping.

The Raman spectra for HA particles (Fig. 1E and F) indicate bands typical of phosphate group vibrations. The band at 425 cm^−1^ corresponds to the O–P–O bending modes, and the band at 588 cm^−1^ represents the *ν*_4_ bending mode of phosphate groups. The band at 957 cm^−1^ is attributed to the *ν*_1_ symmetric phosphate stretching. This did not show any shift in the spectra related to the chemical composition of the materials. This is similar to the behaviour of the 1045 and 1075 cm^−1^, which are associated with *ν*_3_ asymmetric phosphate stretching modes. In the high wavenumber region, the structural OH stretching mode was found at 3572 cm^−1^.^45,46^ The area ratio of the OH band at 3572 cm^−1^ to the phosphate band at 957 cm^−1^ was utilized as an indicator of hydroxyl group concentration^47^ and thus can be used as a measure of dehydroxylation degree^48^ during synthesis. The pure HA group exhibited the lowest area ratio (0.144), indicating a lower relative hydroxyl content, whereas Cu incorporation progressively increased this ratio (1% Cu: 0.170 and 5% Cu: 0.179). This suggests that Cu doping may inhibit dehydroxylation. In the HA group, narrow peaks with high intensity at 3600 cm^−1^ suggest an ordered structure with a set position for the hydroxyl ion.^49^ Additionally, the FWHM of the 3572 cm^−1^ OH band was analyzed to assess structural order and hydrogen-bonding complexity. Pure HA showed the narrowest OH band (FWHM: 8.569 cm^−1^), indicative of a more ordered hydroxyl environment. However, upon Cu doping, the FWHM was significantly broadened (1% Cu: 8.742 cm^−1^, 5% Cu: 8.948 cm^−1^) and the bands showed a significant shift, reflecting increased structural disorder and complexity within the hydroxyl group's hydrogen-bonding network (Table 2). Therefore, these Raman results collectively suggest that Cu incorporation preserves hydroxyl groups within the HA structure while simultaneously inducing structural perturbations and increasing structural disorder, particularly at higher Cu concentrations. In the Raman spectra of the composites (Fig. 1G), the broad band at 3439 cm^−1^ is due to O–H stretching from hydrogel components and water content, while the band at 2933 cm^−1^ is attributed to C–H stretching of the PVA backbone.

**Table 2:** Area ratio and FWHM of HA/Cu-HA composites

Type of HA particles	Area ratio (3572 cm^−1^/957 cm^−1^)	FWHM of the 3572 band
HA (120 °C)	0.144	8.569
1% Cu-HA	0.170	8.742
5% Cu-HA	0.179	8.948

The FTIR spectra for the synthesized composites show characteristic bands that indicate the successful formation of the composite materials (Fig. 1C and D). In the PVA + CPBA hydrogel, the emergence of a band at 1423 cm^−1^ suggests the formation of boronate ester linkages between PVA and CPBA,^50,51^ and the O–H peak at around 3400 cm^−1^ becomes less pronounced, indicating hydroxyl group consumption during crosslinking. The addition of calcium ions further modifies the FTIR spectrum, with a new band appearing at 1620 cm^−1^ suggesting Ca interaction with carboxylate groups, possibly forming calcium carboxylate complexes. The FTIR spectra of the final composites show bands at 3361 cm^−1^ (broad O–H and adsorbed water) and 1619 cm^−1^ (C=O stretching of carboxylate groups). The addition of HA is confirmed by the appearance of a strong band at 607 cm^−1^, corresponding to phosphate bending vibrations typical of HA. Bands at 1016 cm^−1^ (PO_4_^3−^ symmetric stretching) and a slight shift in the O–H stretching band indicate the interaction between HA and the polymer matrix. The spectra of composites with Cu and Sr-HA show distinct changes, including variations in peak intensity associated with phosphate and hydroxyl groups, and the persistence of the 1431 cm^−1^ band, indicating stability of the boronate groups in the presence of metal ions. These spectra suggest that crosslinking within the hydrogel is unaffected by the incorporation of Cu and Sr-HA, but slight phosphate peak shifts suggest possible interactions of the metal ions with the HA particles that influence HA's crystalline structure.

### 3.2 Swelling and degradation properties

The swelling ratios for each group plateaued from 6 h onward, while the composites that contained HA had lower swelling ratios. Under the same pH value, different concentrations of CPBA had no measurable effect on the degradation of composites (SI Fig. 2A and B), but under the same concentrations of CPBA, pH 8.4 of CPBA showed a decreased degradation rate of composites. All groups could remain more than 28 days in SBF (SI Fig. 2C–F).

### 3.3 Injectability and mechanical properties

Injectable rates of the composite were above 80% in all groups (SI Fig. 3J). Their compressive strength was found to be roughly 2 MPa, and there was no significant difference among the groups. As the crystallinity of HA increases, the compressive modulus of the composite also increased (SI Fig. 3I).

### 3.4 Cytotoxicity of optimized composites with hMSCs and macrophages

To ensure clinical biosafety, we deliberately avoided highly toxic traditional chemical crosslinkers (*e.g.*, glutaraldehyde) and harsh solvents (*e.g.*, DMSO). While excessive systemic boron can induce skeletal toxicity, the specific concentration and pH of the CPBA were strictly optimized (SI Fig. 3). Live-dead staining revealed that, as the CPBA concentration decreased, the number of dead cells also decreased (SI Fig. 3A and B). Composites without HA showed a higher number of dead cells compared to those containing HA, and composites prepared at pH 8.4 displayed slightly higher cytotoxicity, as indicated by an increased number of dead cells. However, overall cell viability remained above 70% across all groups, indicating acceptable biocompatibility. Alamar blue assays were used to assess metabolic activity (SI Fig. 3C and E). The results demonstrated that low concentrations of CPBA (3%, 4.5%) enhanced the metabolic activity of hMSCs, while the metabolic activity of macrophages remained comparable across all groups, indicating that the materials were not cytotoxic to each cell type. Pico Green assays (SI Fig. 3D and F) showed no significant differences in dsDNA content among the groups, suggesting consistent cell proliferation across all conditions. Based on the swelling, degradation, and cytotoxicity results, the optimized CPBA (3%, pH = 8.4) was selected for following composite synthesis.

### 3.5 Cell adhesion properties of the composites with different crystalline HA

Alamar blue assays indicated that cells seeded on composites containing HA synthesized at 120 °C exhibited the highest metabolic activity, suggesting improved cell adhesion at this crystallinity level (SI Fig. 3G). Similar results were obtained from Pico Green assays (SI Fig. 3H), supporting the finding that HA synthesized at 120 °C promotes better cell adhesion.

### 3.6 Biocompatibility of Cu and Sr-doped HA composites

**Fig. 2:**
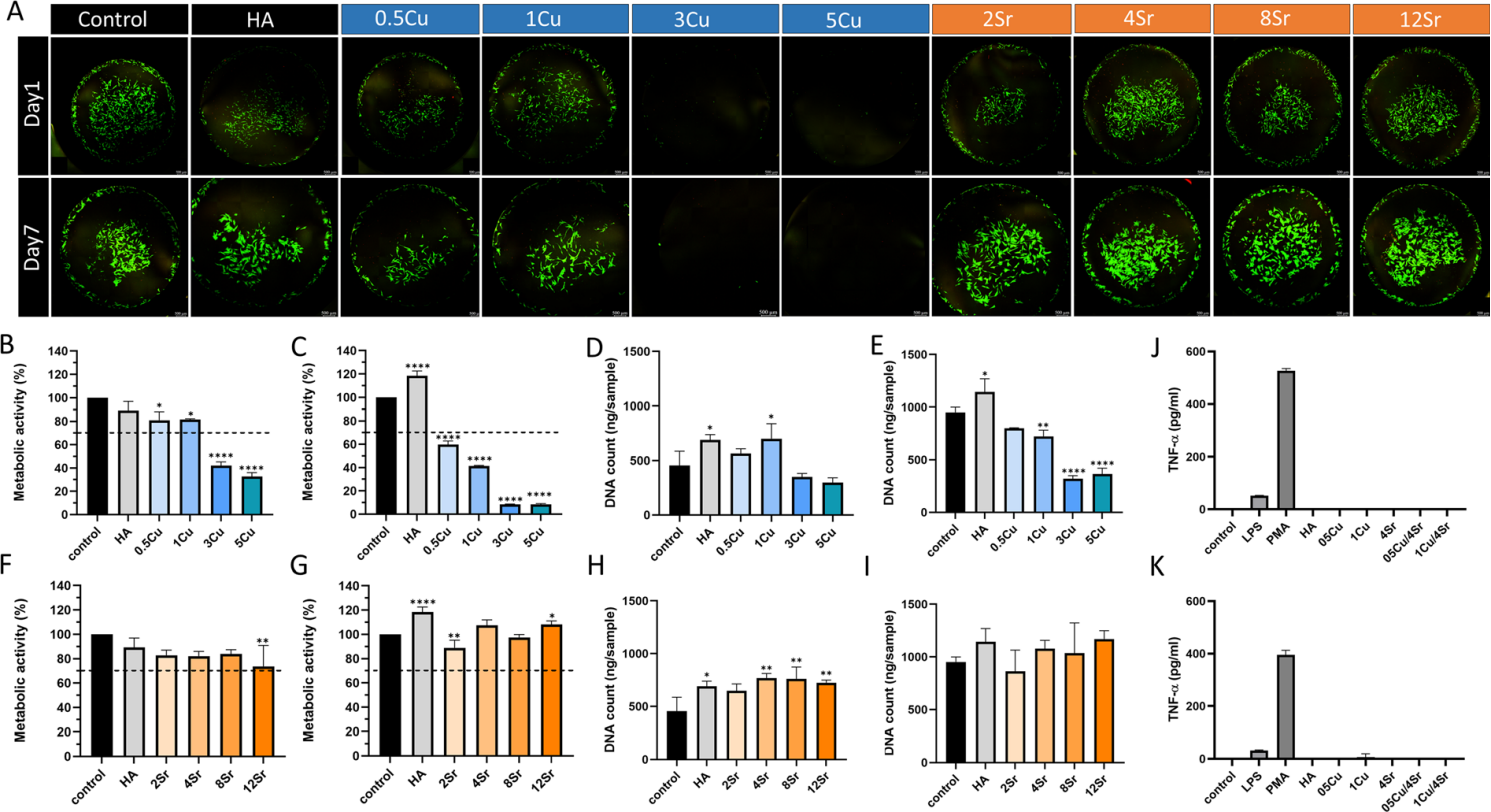
Biocompatibility assessment of the composites mixed with varying concentrations of Cu and Sr-doped HA composites. (A) Representative live-dead staining images of hMSCs at day 1 and day 7; green indicates live cells, red indicates dead cells. Scale bars: 500 µm. Metabolic activity of hMSCs cultured with Cu-HA composites at day 1 (B) and day 7 (C), the dashed line represents the threshold of 70% metabolic activity according to ISO 10993-5:2009. DNA count of hMSCs cultured with Cu-HA composites at day 1 (D) and day 7 (E). Metabolic activity of hMSCs cultured with Sr-HA composites at day 1 (F) and day 7 (G), the dashed line represents the threshold of 70% metabolic activity was used to determine cytotoxicity according to ISO 10993-5:2009. DNA count of hMSCs cultured with Sr-HA composites at day 1 (H) and day 7 (I). (J and K) *Ex vivo* whole blood assays measuring TNF-α levels from two donors exposed to different composites; LPS and PMA are positive controls. *p < 0.05, **p < 0.01, ***p < 0.001, ****p < 0.0001.

The live-dead staining showed that there were no viable cells in the 3% and 5% Cu-HA composites on both day 1 and day 7 (Fig. 2A), indicating significant cytotoxicity at these high concentrations of Cu. Cell proliferation and expansion were observed at day 7 with composites containing lower concentrations of Cu (0.5% and 1%) and all Sr concentrations promoted cell expansion. Alamar blue data indicated that hMSCs' metabolic activity in the 3% and 5% Cu-HA composites were below 70% on day 1, and these values further decreased by day 7, suggesting continued cytotoxicity at these Cu concentrations (Fig. 2B and C). The metabolic activity of cells in 0.5% and 1% Cu-HA composites also decreased after 7 days. In contrast, all Sr-HA composites showed no cytotoxicity on either day 1 or day 7, and cell metabolic activity increased after 7 days, with no evidence of toxicity (Fig. 2F and G). The Pico Green results were consistent with the Alamar Blue data, showing significantly lower dsDNA content in the higher Cu concentration groups (Fig. 2D, E, H and I). The dsDNA content in all groups did not increase significantly after day 7, likely because the cells were seeded at near-confluent densities, limiting further proliferation. The basic whole blood assay was performed to assess TNF-α production in two donors, including two positive controls (LPS and PMA) and a negative control (regular RPMI 1640 medium) to establish baseline cytokine levels (Fig. 2J and K). In the experimental groups, TNF-α production remained at baseline levels, comparable to the negative control, indicating no significant induction of the inflammatory response.

### 3.7 Angiogenic properties of Cu and Sr-doped HA composites

**Fig. 3:**
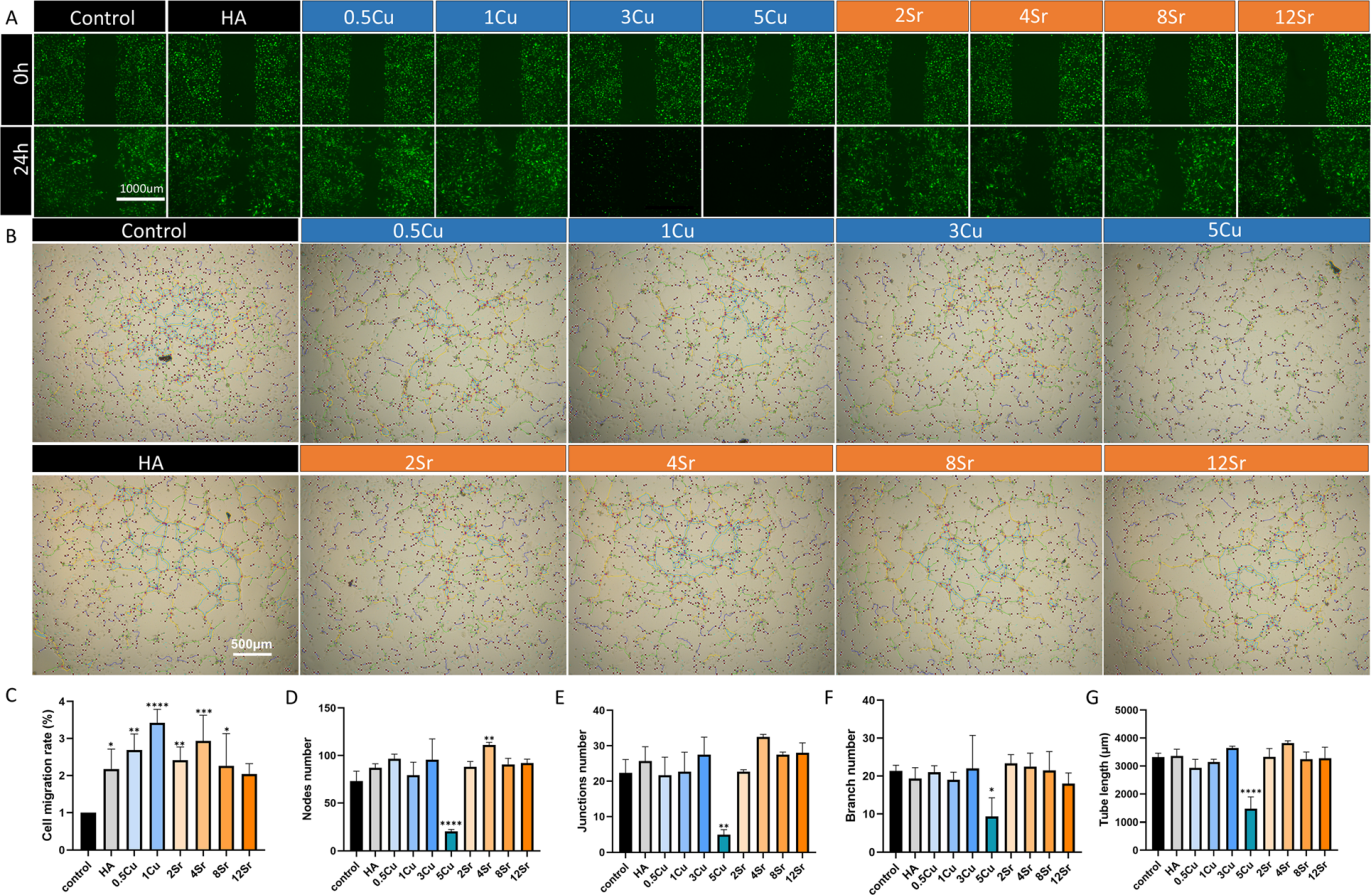
Evaluation of the angiogenic properties of the composites mixed with varying concentrations of Cu and Sr-doped HA composites. (A) Representative images of the migration assay using HUVECs after 0 h and 24 h of incubation with composites. Scale bar: 1000 µm. (B) Representative images of the tube formation assay, analyzed using ImageJ mapping, showing tube networks formed by HUVECs after 6 hours. Scale bar: 500 µm. (C) Quantitative analysis of cell migration rates from the scratch assay. (D–G) Quantitative analysis of tube formation assay parameters: nodes number (D), junctions number (E), branch number (F), and total tube length (G). *p < 0.05, **p < 0.01, ***p < 0.001, ****p < 0.0001.

In the wound healing assay, HUVECs were absent in the 3% and 5% Cu-HA composites after 24 hours, indicating significant cytotoxic effects at these Cu concentrations (Fig. 3A). In contrast, the 1% Cu and 4% Sr-HA composites demonstrated enhanced cell migration at 24 hours compared to the control group (regular EGM-2 medium) (Fig. 3C). The tube formation assay further evaluated the effects of different dopant concentrations on angiogenesis. The results indicate that treatment with varying groups of dopants did not significantly impair the ability of HUVECs to form tube-like structures, except in the 5% Cu-HA composite, where tube formation was notably reduced (Fig. 3B). In the 3% Cu-HA composite, tube formation was observed, indicating that Matrigel might reduce Cu toxicity, thereby enabling higher concentrations of Cu to have a wider range of applications. Quantification of tube formation parameters, including the number of nodes, junctions, branches, and total tube length, showed that the 4% Sr-HA composite had the highest number of nodes and junctions (Fig. 3D–G).

### 3.8 Osteogenic properties of Cu and Sr-doped HA composites

**Fig. 4:**
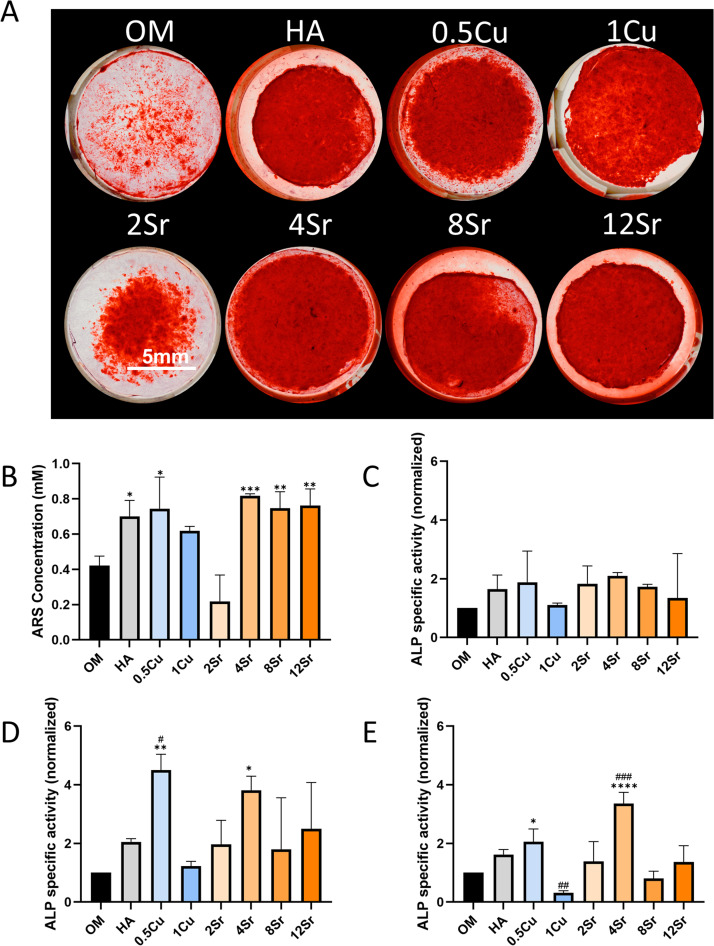
Osteogenic evaluation of the composites mixed with varying concentrations of Cu and Sr-doped HA composites. (A) Representative images of Alizarin Red staining showing calcium deposition at day 21. Scale bar: 5 mm. (B) Quantitative analysis of calcium deposition. ALP activity assays were performed on days 7 (C), 10 (D), and 14 (E). * indicates significant differences compared with the control group (OM), and # indicates significant differences compared with the pure HA composite. (*, #*p* < 0.05; **, ##*p* < 0.01; ***, ###*p* < 0.001).

Due to the observed cytotoxicity of 3% and 5% Cu-HA composites for hMSCs in live-dead staining, these two groups were excluded from further osteogenic experiments. All experimental groups demonstrated considerable calcification, with visible red staining indicating mineralized nodules formation across the samples (Fig. 4A). The 4% Sr-HA composite showed particularly intense staining, suggesting enhanced mineral deposition compared to the other groups. Quantification of Alizarin Red staining revealed that all groups, including those with Sr and Cu-HA composites, exhibited significant calcification compared to the control (regular osteogenic medium), with the 4% Sr-HA composite showing the highest levels of calcification (Fig. 4B). This suggests that Sr at these concentrations effectively enhances the osteogenic potential of the composite material. The ALP activity was normalized against the dsDNA content to account for variations in cell number (Fig. 4C–E). The results showed that 0.5% Cu and 4% Sr-HA composites had significantly higher ALP activity on days 10 and 14 compared to other groups, indicating increased osteogenic differentiation. The elevated ALP activity observed in these groups suggests that the presence of low concentrations of Cu and optimal concentrations of Sr promotes early-stage osteogenic differentiation, which is critical for effective bone regeneration.

### 3.9 Synergistic effect of the co-doped Cu/Sr-HA composites on angiogenic properties

**Fig. 5:**
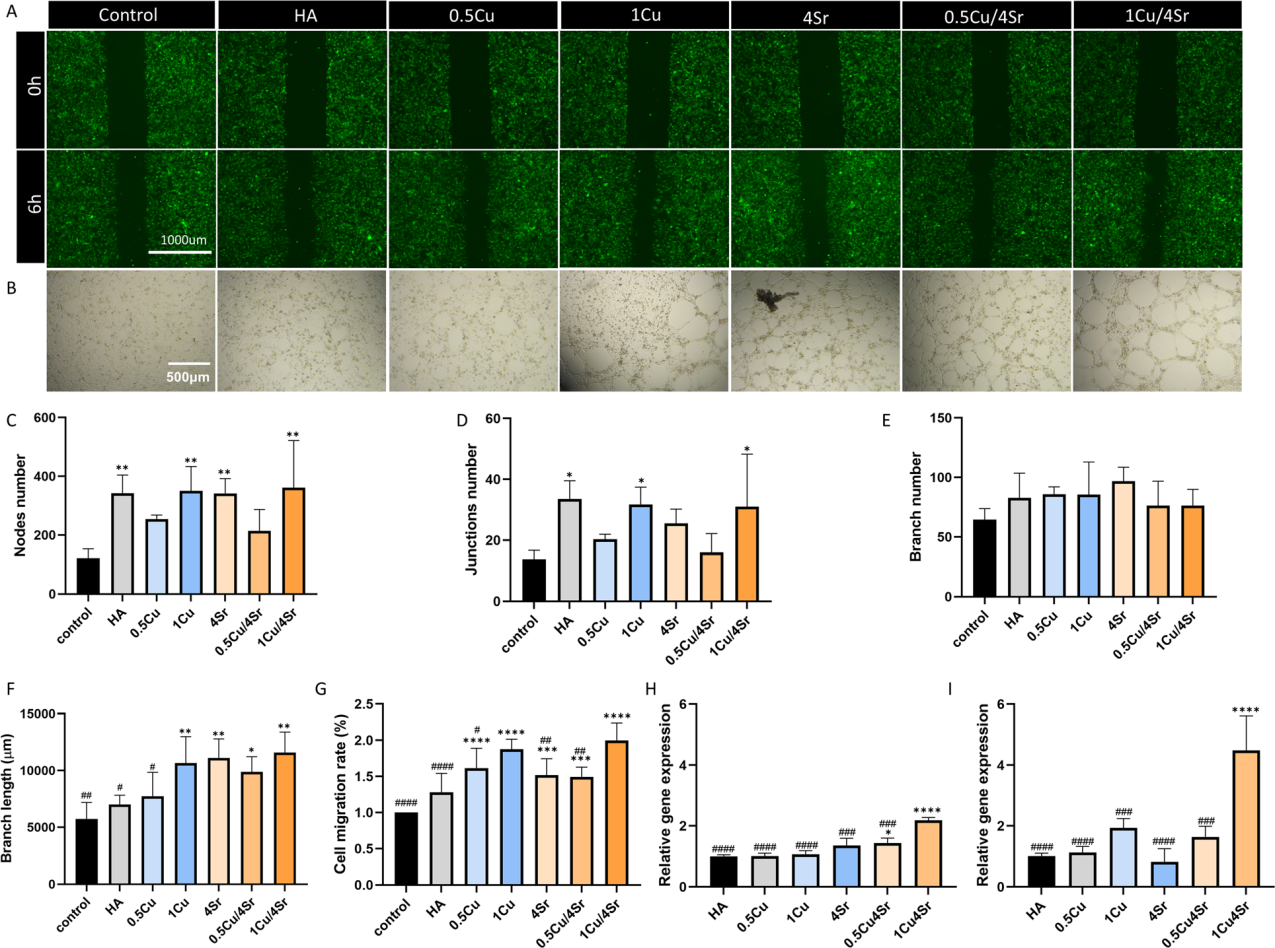
Evaluation of the synergistic angiogenic effects from the composites mixed with varying concentrations of co-doped Cu/Sr-HA composites. (A) Migration assay images of HUVECs at 0 and 6 hours. Scale bar: 1000 µm (B) Representative images from the tube formation assay after 6 hours. Scale bar: 500 µm. (C) Quantification of migration assay. Quantitative analysis of tube formation assay, including the number of nodes (D), junctions (E), branches (F), and total branch length (G). Gene expression analysis (qRT-PCR) of angiogenic markers CD31 (H) and VEGF (I). * indicates significant differences compared with the control group, and # indicates significant differences compared with the 1%Cu/4%Sr-HA composite group. (*, #*p* < 0.05; **, ##*p* < 0.01; ***, ###*p* < 0.001; ****, ####*p* < 0.0001).

Based on the *in vitro* assay results above, 0.5% Cu, 1% Cu, and 4% Sr-HA composites were selected to further investigate the synergy of Cu and Sr in promoting angiogenesis and osteogenesis. In the wound healing assay, cell migration was observed after 6 hours (Fig. 5A). All composite groups showed enhanced migration compared to the control, with the composite with 1% Cu/4% Sr-HA exhibiting the highest migration rate, which was significantly greater than that of the HA, 0.5% Cu, 4% Sr, and 0.5% Cu/4% Sr composites (Fig. 5G). This suggests a synergistic effect of Cu and Sr at optimal concentrations, which promoted endothelial cell migration more effectively than the individual dopants. The tube formation assay showed that HUVECs incubated with the different composites significantly increased capillary structures compared with the control group (Fig. 5B). The 1% Cu/4% Sr-HA composite, in particular, exhibited the longest branch length and significantly higher numbers of nodes and junctions than the HA and 0.5% Cu-HA composites (Fig. 5C–F). qRT-PCR analysis (Fig. 5H and I) was conducted to further evaluate the angiogenic capacity of Cu/Sr-doped composites by examining the expression of angiogenic markers CD31 and VEGF after 14 days of culture. Compared to the control group, the composite groups (0.5% Cu/4% Sr and 1% Cu/4% Sr) demonstrated significantly increased CD31 expression levels, indicating enhanced endothelial cell activity and vascular development. In parallel, VEGF expression was significantly higher in the 1% Cu/4% Sr-HA composite than in all other experimental groups, reinforcing the synergistic angiogenic effect of combining optimal Cu and Sr concentrations.

### 3.10 Synergistic effect of the co-doped Cu/Sr-HA composites on osteogenic properties

**Fig. 6:**
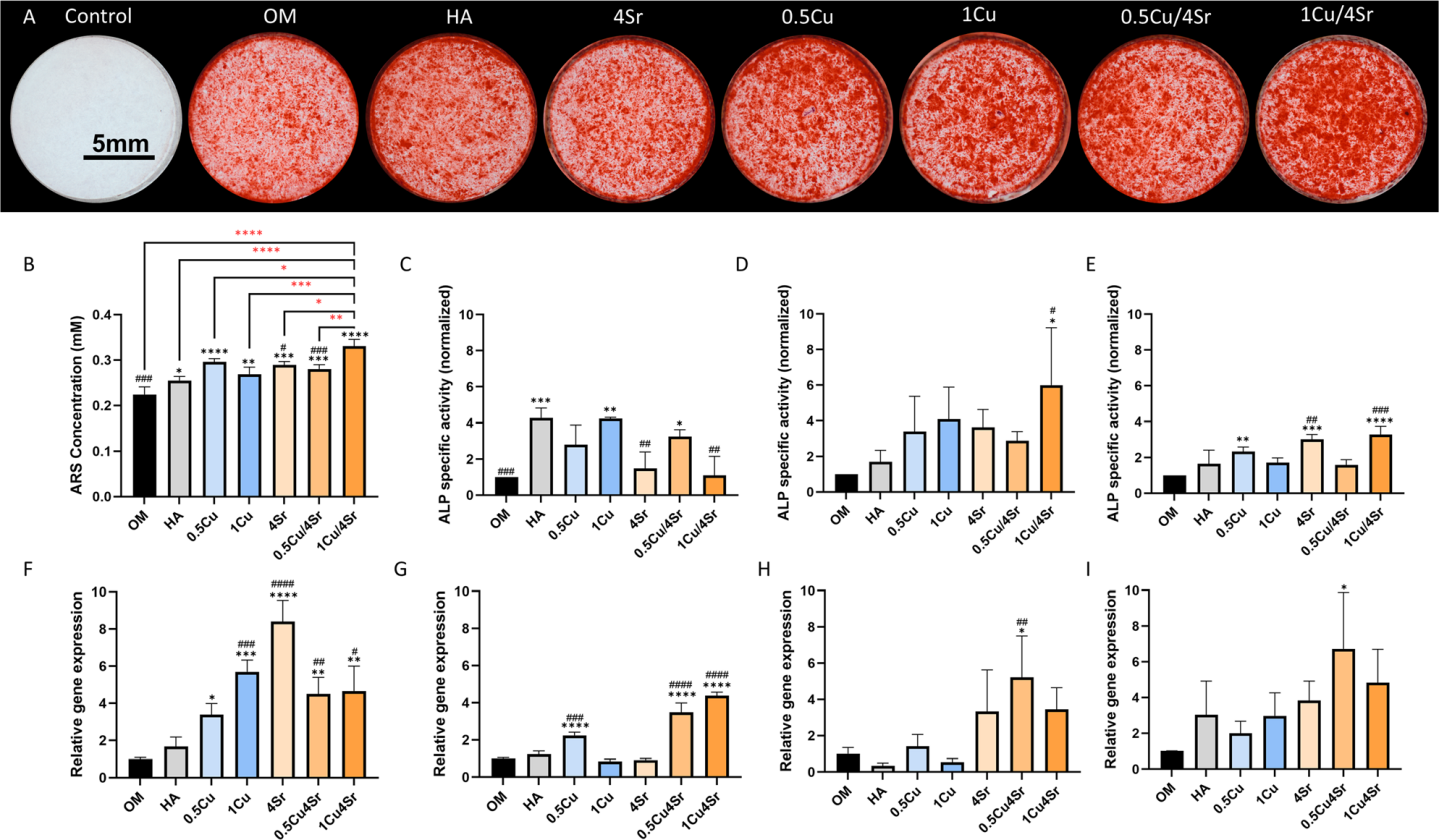
Evaluation of the synergistic osteogenic effects from the composites mixed with varying concentrations of co-doped Cu/Sr-HA composites. (A) Representative images of Alizarin Red staining, indicating calcium deposition. Scale bar: 5 mm. (B) Quantification of calcium deposition. (C–E) Quantitative ALP activity assay at days 7 (C), 10 (D), and 14 (E). Gene expression analysis (qRT-PCR) for osteogenic markers: RUNX2 (F), ALP (G), Col1 (H), and OCN (I). * indicates significant differences compared with the control group (OM), and # indicates significant differences compared with the pure HA composite group (*, #*p* < 0.05; **, ##*p* < 0.01; ***, ###*p* < 0.001; ****, ####*p* < 0.0001).

The *in vitro* osteogenic differentiation potential of Cu- and Sr-co-doped HA composites was evaluated through Alizarin Red staining, ALP activity assays normalized by DNA count, and quantitative qRT-PCR analysis for key osteogenic markers, including RUNX2, ALP, Col1, and OCN. Alizarin Red staining and its quantification performed after 14 days demonstrated evident mineralization across all experimental groups (Fig. 6A and B). The 1% Cu/4% Sr-HA composite showed significantly enhanced calcification compared to other groups, indicating superior osteogenic differentiation and mineral deposition; on day 10, the ALP activity (Fig. 6C–E) in the 1% Cu/4%Sr-HA composite was significantly higher than both the control and HA groups. On day 14, the difference became even more pronounced. This was due to the 1% Cu/4% Sr-HA composite exhibiting significantly elevated ALP activity compared to control, HA, and the 1%Cu-HA composite, emphasizing its superior osteogenic differentiation capabilities. Gene expression analysis *via* qRT-PCR provided additional insights into the molecular pathways underlying osteogenesis. After 14 days of culture, the ALP gene expression pattern showed significant upregulation in both the 0.5% Cu/4% Sr and 1% Cu/4% Sr-HA composites (Fig. 6F–I). Compared with the control group and HA group, RUNX2 expression was significantly upregulated in the rest of the experimental groups. The expression of Col1 and OCN—markers of matrix maturation and late-stage osteogenesis, respectively—was elevated in the 0.5% Cu/4% Sr and 1% Cu/4% Sr-HA composites. However, the increase observed in the 1% Cu/4% Sr-HA composite was not statistically significant.

To verify whether the dual-ion strategy yields true mathematical synergism rather than merely additive enhancement, we quantitatively evaluated the expected additive effects for key biological markers (SI Table S1). By defining the net biological effect (Δ*E*) as the difference between the experimental group and the HA control baseline, the theoretical additive effect of the single-doped components was calculated (Δ*E*_Expected_ = Δ*E*_Cu_ + Δ*E*_Sr_).^52^ Our quantitative assessment revealed a nuanced multi-stage regulation: true synergism (Δ*E*_Observed_ > Δ*E*_Expected_) was strongly evident in the gene expression of crucial angiogenic initiators (VEGF, CD31) and osteogenic drivers (ALP gene, OCN gene), as well as in the final mineralization. Conversely, mid-stage activities, such as Col1 expression and 10-day ALP enzyme activity, exhibited robust additive effects.

## 4. Discussion

Bone defects, particularly those of critical size, remain a major clinical challenge.^4^ There is an urgent need for artificial bone grafting materials that are biodegradable, immunocompatible, and capable of promoting osteogenesis.^53^ We developed and optimized an injectable dual-network hydrogel composed of PVA and SA, incorporating hydrothermally synthesized HA doped with Cu and Sr. This system aims to simultaneously promote osteogenesis and angiogenesis while maintaining biodegradation and mechanical stability. Our main findings indicate that HA synthesized at 120 °C exhibited moderate crystallinity, offering an optimal balance between mechanical strength and cell adhesion. Low concentrations of Cu (0.5–1%) and moderate levels of Sr (4%) preserved cell viability and suppressed inflammatory responses, whereas higher concentrations of Cu were cytotoxic. Moreover, single Cu and Sr-HA composites enhanced endothelial cell migration and osteogenic activity. Notably, co-doping Cu and Sr-HA composites yielded a synergistic effect, further enhancing both angiogenic and osteogenic responses. Biologically, bone regeneration follows a coordinated temporal sequence in which angiogenesis precedes and tightly couples with osteogenesis. The initial hydrolysis of the dynamic hydrogel network (boronate ester and ionic crosslinks) provides an early, moderate release of superficially associated ions, whereas the subsequent degradation of the 120 °C crystalline HA particles supplies a longer-term, sustained ionic reservoir. Importantly, recent crystallographic insights, such as those of Kis *et al.*,^42^ show that native bone mineral is composed of monoclinic calcium phosphate. Our modulation of synthetic HA crystallinity was therefore not aimed at atomic-level mimicry of native bone, but at tuning surface properties and protein adsorption, as supported by our adhesion assays. HA crystallinity controlled by hydrothermal synthesis temperature significantly influenced biological and mechanical properties.^54^ The degree of crystallinity directly shapes the nanotopography, surface energy, and density of active crystal defect sites, which together govern the initial adsorption and conformational presentation of adhesive serum proteins.^55,56^ By tuning crystallinity, we therefore optimize the nascent bio-interface, providing the mechanotransductive foundation on which Cu/Sr-mediated signalling can drive subsequent osteogenic differentiation. We identified 120 °C as the optimal temperature, effectively balancing crystallinity to support cellular activities without sacrificing mechanical integrity. This aligns with Balasundaram *et al.*,^38^ who reported superior osteoblast responses to low-crystalline HA. EDX, FTIR, and Raman spectra confirmed that uniform incorporation of Cu and Sr within the HA structure and hydrogel composite was achieved. As the pH-dependent crosslinker, CPBA is favored to form a boronate linkage at higher pH value.^57,58^ By optimizing the concentration and pH of CPBA (3%, pH = 8.4), a balance was achieved between the composite stability and its biocompatibility. Regarding cytotoxicity, we observed significant reductions in cell viability at higher Cu concentrations (≥3%), aligning with Li *et al.*,^28^ who reported Cu-induced cytotoxicity at similar thresholds. While Sr doping showed a broader therapeutic window, consistent with findings by Gomes Luz *et al.*,^59^ supporting its versatility in higher-dose applications. Optimal concentrations (Cu: 0.5–1%, Sr: 4%) enhanced angiogenic and osteogenic potential; these concentrations differ slightly from previous studies and may be due to different loading systems.^27,60^ Due to the different functions and mechanisms of Cu and Sr in osteogenesis and angiogenesis, we considered incorporating Cu and Sr into HA to verify whether the synergistic effect can be promoted more effectively. This procedure revealed that co-doping HA with 1% Cu and 4% Sr composite substantially enhanced endothelial migration and VEGF, CD31 genes' expression, corroborating prior observation,^61^ and co-doping also markedly elevated osteogenic markers (ALP, OCN, Col1) and mineralization beyond single-ion doping effects. The TNF-α ELISA result indicated that no composites caused any pronounced pro-inflammatory response under *ex vivo* conditions, highlighting their potential for clinical translation.

While previous studies have explored Cu/Sr co-doping in ceramics, glasses, or metallic substrates, few have systematically examined how HA crystallinity, ion concentration, and delivery format collectively influence biological outcomes.^61–63^ Our study addresses these gaps by precisely modulating HA crystallinity *via* hydrothermal synthesis. It also incorporates optimally doped HA (0.5–1% Cu, 4% Sr) into a biodegradable dual-network hydrogel system. We found that Cu above 1% induced significant cytotoxicity in our system, consistent with earlier reports;^27^ however, Matrigel notably reduced this effect in endothelial assays. Another finding is unexpectedly low ALP activity in 1% Cu despite strong mineral deposition. This likely reflects the temporal dissociation between ALP and late-stage mineralization, with transient 1% Cu overexposure initially inhibiting ALP but still promoting osteogenesis *via* pathways such as Wnt/β-catenin or VEGF indirectly.^64,65^ In contrast, Li *et al.*^28^ concluded that Cu possibly limits the osteogenic differentiation of MSCs and restrains *in vivo* bone formation through the Runx2 pathway; however, this could also be due to the high concentration of Cu (5 µmol L^−1^) used. This reveals the complexity of Cu's role during the process of bone regeneration and the need to carefully choose Cu concentration. Similarly, although Cu/Sr-HA composites enhanced overall osteogenesis, the 1% Cu/4% Sr-HA composite showed lower RUNX2 expression than the Sr-only group at day 14, likely because the synergistic effect accelerated osteogenic progression, leading to earlier RUNX2 peaking and subsequent downregulation. This dynamic pattern was also observed in previous studies.^66,67^ These findings offer new mechanistic insights into how Cu/Sr-HA composites not only promote osteogenesis but also modulate the temporal dynamics of osteogenic differentiation. However, as advanced drug-delivery strategies emerge, introducing spatially patterned Cu/Sr domains is a particularly promising concept for next-generation spatiotemporal bone scaffolds.^68^

Despite these exciting results, several limitations should be acknowledged. First, only a single HA concentration (30%wt) has been evaluated, as our preliminary trials indicated that HA loadings exceeding 40%wt compromise homogeneous mixing and handleability of the hydrogel; however, different HA loadings could influence crosslinking, mechanical properties, degradation behaviors, and cell compatibility. Increased HA content also compressed hydrogel pores, potentially restricting swelling and cell infiltration, suggesting that future studies should investigate composite porosity and internal structure. Second, while moderate crystallinity (120 °C HA synthesis) performed effectively, and optimized CPBA (3%, pH = 8.4) maintained the maximum biocompatibility without affecting degradation *in vitro*, adjustments may be necessary to ensure optimal performance under varying mechanical loads and defect geometries *in vivo*. It should be explicitly noted that with an initial compressive modulus of approximately 2 MPa, this injectable hydrogel is deliberately designed for non-load-bearing bone defects (*e.g.*, filling contained cavities) rather than segmental load-bearing applications. Although our structural data (XRD/EDS) suggest a sustained, congruent dissolution mechanism from the HA lattice that prevents cytotoxic burst release, absolute quantification of the real-time actual release kinetics of and *via* inductively coupled plasma mass spectrometry was not performed; precise pharmacokinetic profiling remains an essential next step. Furthermore, the differences in ALP activity *versus* final mineralization outcomes for certain Cu concentrations (*e.g.*, 1% Cu) suggest that additional mechanistic studies are warranted to clarify how Cu influences each phase of osteogenic differentiation, and for gene expression studies, time points should be included both early (*e.g.*, 7 days) and later (*e.g.*, 21 days). And *in vitro* supernatant extraction assays cannot fully capture dynamic surface topographies and 3D local concentration gradients, highlighting the need for future *in vivo* defect models to elucidate the coupled physical-chemical interactions during tissue regeneration. Although our *ex vivo* assay indicated minimal inflammatory response, long-term *in vivo* studies are necessary to confirm chronic biocompatibility and evaluate the composites' performance under physiological conditions. Finally, growth factors such as BMP-2 or VEGF could be incorporated using strategies such as physical entrapment within the hydrogel matrix, or encapsulation in biodegradable microspheres co-loaded into the composite; these approaches would enable localized, sustained release that may further enhance the clinical efficacy of the hydrogel composite, particularly in accelerating bone regeneration, improving vascularization for critical-size defects.

Building on these findings, several directions for future research are proposed. First, a critical-sized femoral defect model in rats with two time points (four and eight weeks) is needed to validate how Cu/Sr co-doping influences bone formation and neovascularization *in vivo*.^69,70^ For instance, by changing the concentration of Cu (such as a lower Cu concentration for more bone formation or a higher Cu concentration for angiogenesis), some highly bioactive materials can be applied to neutralize Cu cytotoxicity. Second, exploring the incorporation of additional bioactive ions (*e.g.*, Mg, Zn) or growth factors may further enhance the regenerative outcomes through synergistic effects. Given the flexibility of our hydrogel system, drug-loaded formulations can be developed in various formats, such as injectable pastes or coatings, to address specific clinical needs. For minimally invasive procedures, a double-barrel syringe with a Y-junction delivers the fast-reacting precursors in a fluid state. Alternatively, for clinical scenarios requiring specific geometric implants, the composite can be fully crosslinked *in vitro* and subsequently punched or molded into customized macroscopic shapes prior to surgical insertion. Third, depending on the different mechanical properties required for different clinical applications, the hydrogel cross-linking density or the coupling of the composite with other polymers or ceramics can be adjusted to improve load-bearing capacity. Last but not least, inspired by recent advances in immunotherapy,^71^ composites could also be engineered to modulate immune responses, such as guiding macrophage polarization (*e.g.*, from M0 to M2), thereby enhancing bone regeneration and tissue integration. The injectable and moldable composite makes it particularly suitable for minimally invasive procedures and irregular bone defects. These include the treatment of critical-sized long bone defects, craniofacial reconstruction, spinal fusion graft enhancement, and post-tumor resection defect filling, while the large-scale manufacturability of the composite system should be evaluated. Notably, we think the composite may also serve as a potential alternative to non-biodegradable Polymethyl methacrylate (PMMA) spacers in the Masquelet technique, which is widely used for managing large bone defects;^72,73^ however, future animal studies are warranted to assess its ability to induce membrane formation and sufficient structural integrity.

## 5. Conclusion

This study demonstrates that hydrothermally synthesized HA can be successfully incorporated into a dual-network PVA/SA hydrogel, and the synthetic temperature can control the HA's crystallinity to alter the composite's mechanical properties and biological activity. By carefully tuning the concentrations of Cu and Sr within the HA structure, we achieved a synergistic effect on both angiogenesis and osteogenesis *in vitro*. Low-to-moderate Cu concentrations (0.5–1%) and moderate Sr concentrations (4%) proved particularly beneficial, whereas exceeding 3% Cu led to cytotoxic outcomes. Mechanistic investigations revealed that distinct stages of osteogenic differentiation, including gene and ALP expression, as well as mineralized nodule formation, exhibit different sensitivities to Cu levels, highlighting the need for precise dose optimization. *Ex vivo* TNF-α assays suggested that these composites do not cause significant inflammatory responses. Our findings highlight the importance of balancing crystallinity, ion doping, and polymer crosslinking within HA-based composites to achieve a dual-purpose system that promotes rapid vascularization and robust bone formation. Future work on *in vivo* validation, customization, and functionalization with additional bioactive agents could advance this approach toward clinical application in critical-size or large bone defect repair.

## Conflicts of interest

The authors declare no conflict of interest.

## Supplementary Material

RA-016-D6RA01689H-s001

## Data Availability

The data supporting the findings of this study are available within the article and its supplementary information (SI). Supplementary information is available. See DOI: https://doi.org/10.1039/d6ra01689h.
