# Supplementary material for: Harnessing copper–strontium synergy in an injectable composite for multi-pathway bone regeneration
Source: RSC Adv. 2026 May 13;16(28):25292–308. doi: 10.1039/d6ra01689h (PMC13169506; doi:10.1039/d6ra01689h)
Supplement: RA-016-D6RA01689H-s001 [file RA-016-D6RA01689H-s001.pdf]

Supplementary Information (SI) for RSC Advances.
This journal is © The Royal Society of Chemistry 2026

Harnessing Copper–Strontium Synergy in an Injectable Composite for Multi-Pathway Bone Regeneration

Zhengqing Zhu[1], Azin Khodaei[1], Helen E. King[2,3], Harrie Weinans[1], Saber Amin Yavari[1,4,*]

1. Department of Orthopedics, University Medical Center Utrecht, 3508GA Utrecht, The Netherlands

2. MARUM, Faculty of Geosciences, University of Bremen, 28359 Bremen, Germany

3. Department of Earth Sciences, Faculty of Geosciences, Utrecht University, 3584CB Utrecht, The Netherlands

4. Regenerative Medicine Centre Utrecht, Utrecht University, 3508GA Utrecht, The Netherlands

***Corresponding author:**

Dr. Saber Amin Yavari

Department of Orthopedics, University Medical Center Utrecht

Utrecht 3508GA

The Netherlands

Tel: +31 (0)88 75 56481

Fax: +31 (0)30 25 106 38

Email: S.AminYavari@umcutrecht.nl, Saber.aminyavari@gmail.com

1. Methods

1.1 Injectability test

The quantitative injectability of the composites containing synthetic HA (80°C, 120°C, 160°C) was evaluated using a customized dual-syringe extrusion method. Briefly, Solution A, Solution B, and the specific HA particles were homogeneously mixed and loaded into one syringe of a 1 mL double syringe. Solution C and $CaCl_2$ were loaded into the other syringe. The two syringes were equipped with a standard Y-junction static mixing nozzle to form the complete delivery apparatus. All tests were performed at room temperature. The fully loaded dual-syringe apparatus was initially weighed (W_1) and then fixed onto a stable vertical holder. A standardized 5 kg compressive load was mounted vertically on top of the plunger to simulate a consistent maximum manual injection force. The load was applied until the plunger completely stopped moving. After the injection process, the syringe apparatus with the residual unexpelled gel was weighed again (W_2). The mass of the completely empty dual-syringe and nozzle set (W_0) was predetermined.

The injectability coefficient (%) was rigorously calculated based on the precise mass of the expelled hydrogel relative to the initial loaded hydrogel mass, according to the following equation:

$$Injectability(\%) = \frac{W1 - W2}{W1 - W0} \times 100\%$$

1.2 Dynamic Mechanical Analyzers

A dynamic mechanical analysis (DMA, Discovery Hybrid Rheometer HR-2, USA) machine was used to measure the mechanical properties of the composites through a compression test. After soaking the lyophilized composites (6mm*3mm) in Phosphate-Buffered Saline (PBS) for 4 hours, the compression test was performed using a Ramp force of 1.00N/min up to 18.00N at room temperature. The elastic modulus in compression was determined by reporting the average slope of the stress-strain curve in the linear elastic zone (10 to 20%).

1.3 Swelling and degradation tests

As a crosslinker, different concentrations and pH of CPBA can influence the characteristics of the composites, thus need to be optimized for the composites' swelling, degradation, and bioactivity behaviour(1,2) A simulated body fluid (SBF)(3) was prepared based on a previously reported protocol and was used to investigate the swelling and degradation rates of composites. In short, based on the concentration (3%, 4.5%, 6% CPBA), pH (7.4, 8.4) and value of CPBA with or without HA, the dried composites (6mm*3mm) were weighed and incubated in SBF at 37°C for 28 days in total; the surface moisture of the samples was removed gently by wiping them with absorbent tissue before weighing them at different time points. The weight/volume ratio of biomaterial/SBF was adjusted to 1g/10ml based on ISO 10993-13.

1.4 Calculation of Synergistic and Additive Effects (Synergy Index)

To quantitatively determine the interaction between Cu and Sr in the co-doped hydrogel composites, the biological responses were evaluated using an additive effect model. The net biological effect (ΔE) for each experimental condition was first determined by subtracting the baseline value of the metal-free control group (HA control). The calculations were performed as follows:

Net Effect of Single Elements:

$$\Delta E_{Cu} = E_{Sr} - E_{Control}$$

$$\Delta E_{Sr} = E_{Sr} - E_{Control}$$

Expected Additive Effect: The theoretical additive effect of the co-doping system was defined as the sum of the individual net effects:

$$\Delta E_{Expected} = \Delta E_{Cu} + \Delta E_{Sr}$$

Observed Net Effect: The actual observed effect of the co-doped group was calculated as:

$$\Delta E_{Observed} = E_{Cu/Sr} - E_{Control}$$

Synergy Index (SI): To standardize the evaluation, a Synergy Index (SI) was calculated as the ratio of the actual net effect to the expected additive effect:

$$SI = \Delta E_{Observed} / \Delta E_{Expected}$$

Interpretation Criteria: The interaction was defined based on the SI value:

Synergism: SI > 1, indicating that the combined effect is greater than the sum of the individual effects.

Additive Effect: SI =1, indicating that the combined effect equals the expected sum of individual effects.

Antagonism: SI < 1, indicating that the combined effect is less than the expected sum.

Supplementary Figure 1

A

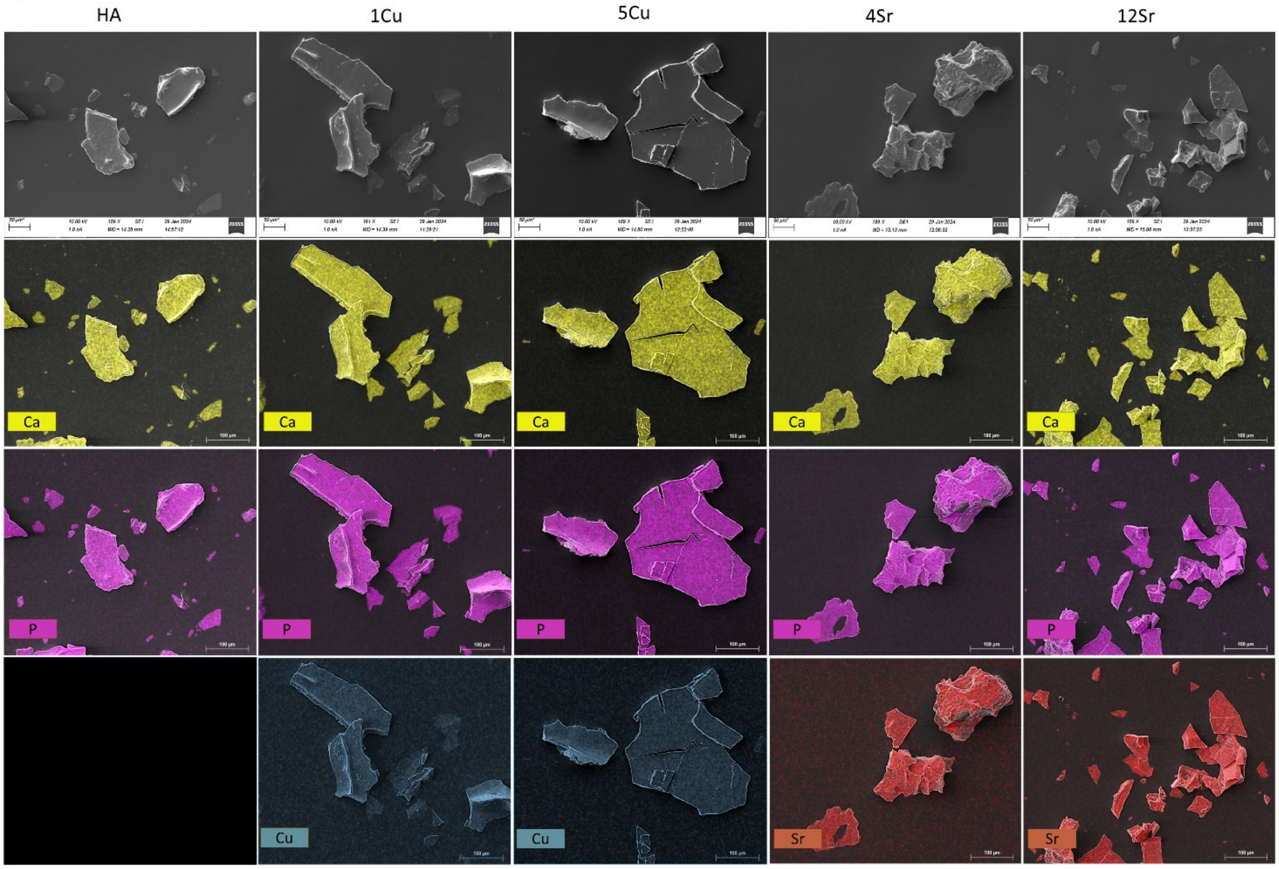

Supplementary Figure 1 A. EDX elemental mapping of HA particles doped with Cu and Sr. Scale bars: 100 µm.

Supplementary Figure 2

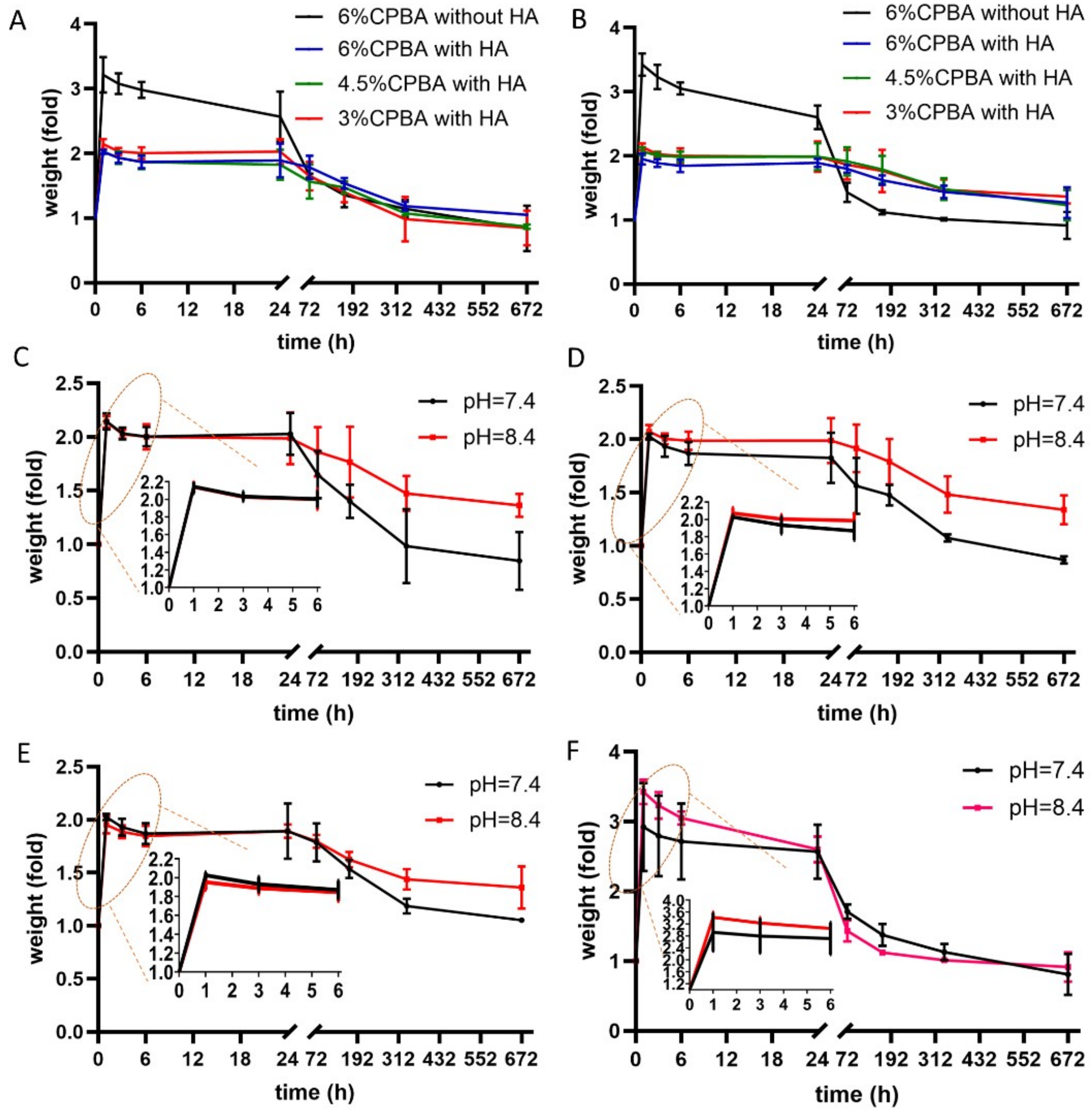

Supplementary Figure 2. Swelling and degradation behavior of the composites with varying CPBA concentrations and pH values. Swelling and degradation profiles of composites synthesized at different CPBA concentrations (3%, 4.5%, 6%) at (A) pH 7.4 and (B) pH 8.4. (C-F) Comparison of swelling and degradation behavior of composites between pH 7.4 and pH 8.4 at constant CPBA concentrations: (C) 3% CPBA, (D) 4.5% CPBA, (E) 6% CPBA, and (F) 6% CPBA without HA. Insets highlight swelling behavior within the initial 6-hour period (n = 3).

Supplementary Figure 3

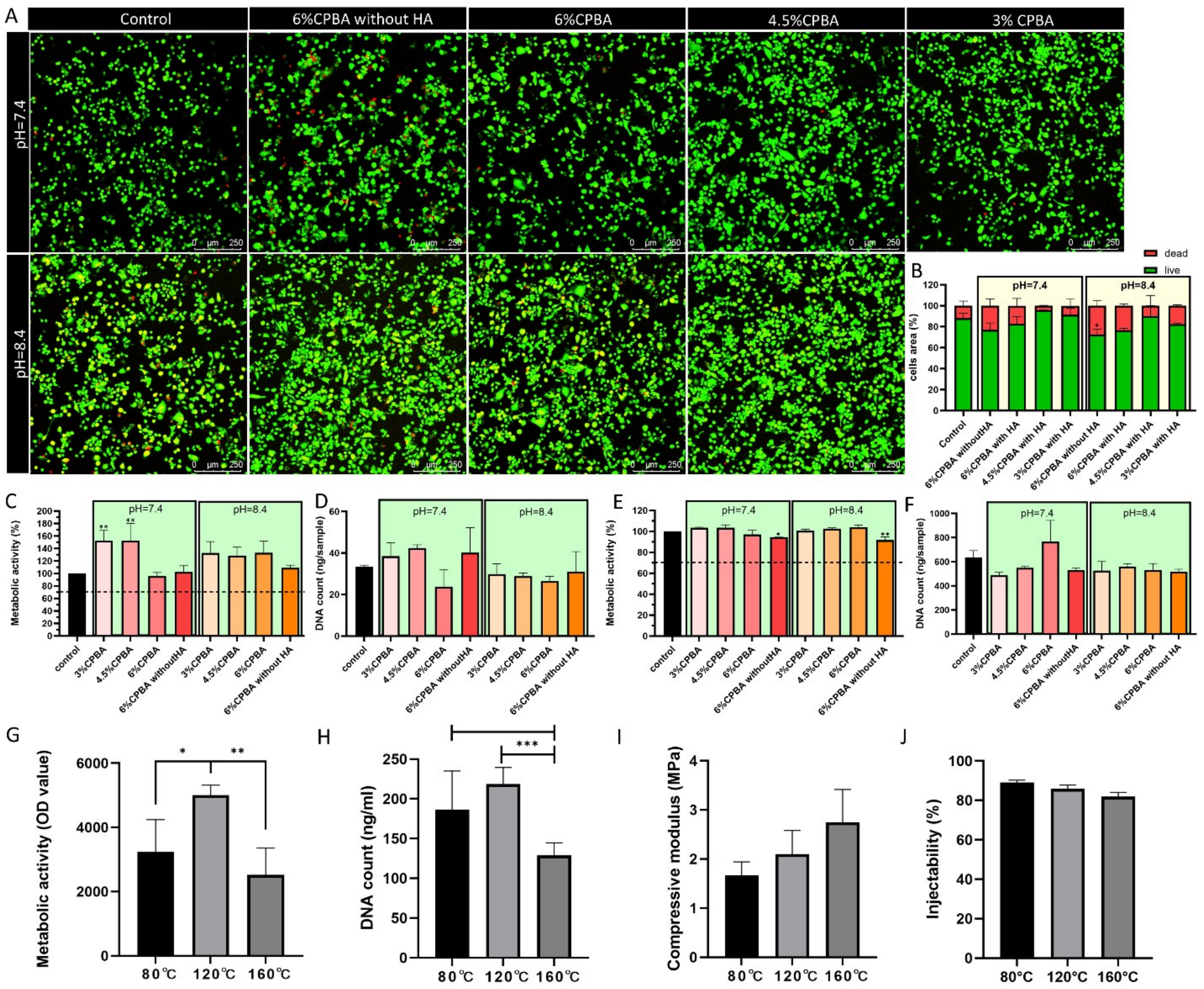

Supplementary Figure 3. Optimization of composite biocompatibility, cell attachment, mechanical properties, and injectability. (A) Representative live-dead staining images of composites prepared with varying CPBA concentrations (3%, 4.5%, 6%) and pH values (7.4 and 8.4); viable cells are stained green and dead cells red. Scale bars: 250 μm. (B) Quantification of live and dead cell percentages. (C-D) Metabolic activity and DNA count of hMSCs cultured with composites. (E-F) Metabolic activity and DNA count of macrophages cultured with composites. (G-H) Metabolic activity and DNA count of hMSCs attached to composites containing HA synthesized at different temperatures (80°C, 120°C, 160°C). (I) Compressive modulus of composites with HA synthesized at different temperatures. (J) Injectability (%) of composites with HA synthesized at different temperatures. *p<0.05, **p<0.01, ***p<0.001.

Supplementary Figure 4

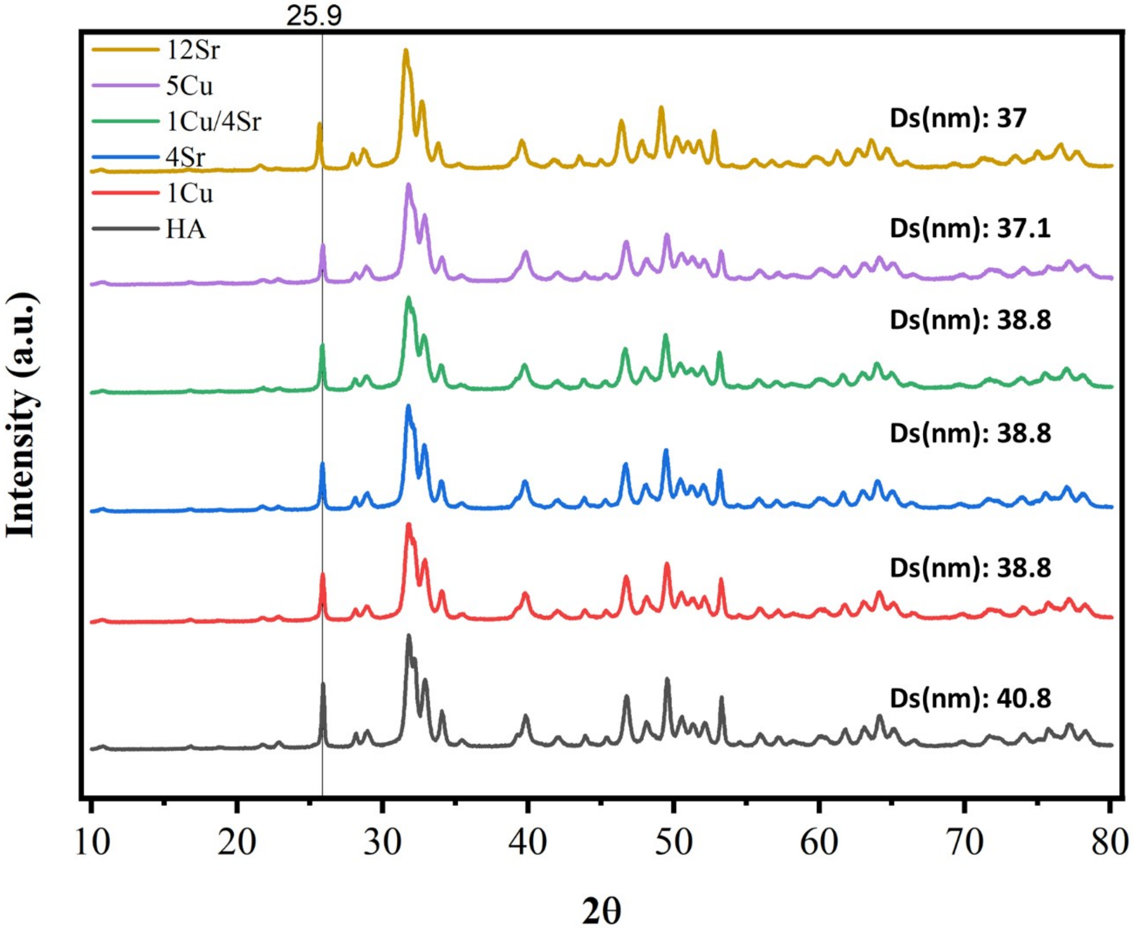

Supplementary Figure S4. X-ray diffraction (XRD) patterns of pure hydroxyapatite (HA) and various single-doped (1%Cu, 5%Cu, 4%Sr, 12%Sr) and co-doped (1%Cu/4%Sr) HA nanoparticles synthesized hydrothermally at 120°C. The vertical gray line marks the standard position of the (002) diffraction plane at 2θ = 25.9°.

Table S1. Quantitative assessment of synergistic effects between Cu and Sr in the 1%Cu/4%Sr-HA composite based on expected additive effects.

Aspects of angiogenesis or osteogenesis	Net Effect of 1Cu (ΔE_{1Cu})	Net Effect of 4Sr (ΔE_{4Sr})	Expected Additive Effect (ΔE_{1Cu} + ΔE_{4Sr})	Observed Net Effect of 1Cu/4Sr ($\Delta E_{1Cu/4Sr}$)	Synergy Index (SI)
CD31 expression	0.073333	0.353333	0.426666	1.183333	2.77344105
VEGF expression	0.93333	-0.185	0.74833	3.485	4.65703633
Cell migration rate	0.467467	0.237102	0.704569	0.715982	1.01619856
ALP expression	-0.38573	-0.30631	-0.69204	3.148302	-4.5493064
Col1 expression	0.193333	2.98	3.173333	3.1	0.97689086
OCN expression	-0.05667	0.803333	0.746663	1.81	2.42411905
ALP 10 days	2.383957	1.946048	4.330005	4.301457	0.99340694
ALP 14 days	0.055386	1.367304	1.42269	1.629201	1.1451553
Alizarin red quantification	0.014161	0.0034819	0.017643	0.064475	3.65442385

Note: net effect ΔE is calculated by subtracting the baseline value of the HA control group. "Synergistic" is defined as an observed net effect that exceeds the expected additive effect.

1. Zhao Y, Li M, Liu B, et al. Ultra-tough injectable cytocompatible hydrogel for 3D cell culture and cartilage repair. *J Mater Chem B*. 2018;6(9):1351-1358. doi:10.1039/C7TB03177G

2. Marco-Dufort B, Tibbitt MW. Design of moldable hydrogels for biomedical applications using dynamic covalent boronic esters. *Mater Today Chem*. 2019;12:16-33. doi:10.1016/j.mtchem.2018.12.001

3. Kokubo T, Takadama H. How useful is SBF in predicting in vivo bone bioactivity? *Biomaterials*. 2006;27(15):2907-2915. doi:10.1016/j.biomaterials.2006.01.017
